# Toward Efficient and Reliable Chemical Upgrading Using Solid Oxide Electrochemical Reactors, Mechanisms, Challenges, and Design Principles

**DOI:** 10.1002/smll.73272

**Published:** 2026-04-07

**Authors:** Nai Shi, Yun Xie, Moses Oludayo Tadé, Zongping Shao

**Affiliations:** ^1^ Curtin Center For Advanced Energy Materials and Technologies WA School of Mines (WASM) Curtin University Perth Western Australia Australia; ^2^ Department of Energy Conversion and Storage Technical University of Denmark Lyngby Denmark

**Keywords:** solid oxide electrochemical reactor, chemical upgrade, olefin synthesis, CO_2_ reduction, electrode design, efficiency, stability

## Abstract

Solid oxide electrochemical reactors (SOERs) offer a compelling pathway for upgrading feedstocks into value‐added chemicals using renewable electricity, in which electrode reaction kinetics can be precisely regulated by external electricity to surpass reaction thermodynamic limitations. However, the practical deployment of SOERs remains constrained by low product yields and instability. Existing studies have largely focused on isolated material innovations and have reported scattered performance data under seemingly similar conditions, lacking an integrated perspective that bridges material design, device engineering, and electrochemical coupling. Here, we present a comprehensive review of both protonic and oxygen‐ion‐conducting SOERs for chemical synthesis. We first outline reaction mechanisms and cell configurations across key reactions, including cathodic CO_2_ upgrading, anodic methane coupling, alkane‐to‐olefin conversion, and their hybrid pathways, establishing a foundation for next‐generation material development. We then summarize the critical factors governing conversion efficiency, product selectivity, and operation stability from both electrochemical and catalytic perspectives. Subsequently, recent advances in electrode development for enhancing electrochemical performance and product yields are summarized and compared. Finally, future opportunities and research directions are outlined to accelerate the commercial translation of SOER technologies. This review provides a framework for understanding complex SOER‐driven chemical upgrading and offers guidance for its development.

## Graphical abstract

Solid oxide electrochemical reactors (SOERs) offer a compelling pathway for coupling renewable electricity with chemical manufacturing, but their real application is still limited by low efficiency and instability. This review systematically summarizes SOER reaction mechanisms, performance‐limiting factors, and recent progress toward high‐efficiency and stable operation.

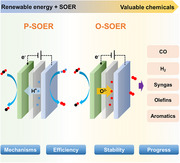

## 1 Introduction

Rapid industrialization has increased atmospheric CO_2_ concentrations from 280 ppm in 1750 to over 430 ppm in 2026 [1], making it a major cause of climate change and biodiversity loss. Meanwhile, the global pursuit of carbon neutrality has accelerated the deployment of renewable energy technologies. However, renewable electricity sources such as wind, hydro, and solar power are inherently intermittent, creating an urgent need for efficient systems capable of converting these fluctuating energies together with chemicals into value‐added chemical products [2, 3]. Among various technologies, the electrochemical upgrading of chemicals into value‐added products is particularly promising due to its high efficiency and compact size. A major advantage of electrochemical synthesis over conventional thermal catalytic systems lies in their controllable reactions, which can be precisely tuned by adjusting the external currents, potentially overcoming thermodynamic limitations. Although low‐temperature electrochemical devices have achieved remarkable success in water electrolysis [4], CO_2_ reduction [5], and chemical synthesis [6], their inherently slow reaction kinetics necessitate high electrical input and the use of precious catalysts to maintain acceptable efficiency, which hinders their commercial viability.

Solid oxide electrochemical reactors (SOERs) offer significant advantages, including high efficiency, fast reaction kinetics, operational stability, and low cost. Featuring an all‐solid‐state architecture and operating at elevated temperatures (400–850°C), SOERs can seamlessly integrate renewable electricity and waste heat to upgrade low‐value feedstocks (e.g., CO_2_, H_2_O, alkanes, and N_2_) into value‐added products such as CO, H_2_, olefins, and NH_3_ [7, 8, 9, 10, 11]. SOERs can also be coupled with thermocatalytic processes, such as biomass pyrolysis, to achieve deoxygenation and hydrogenation and obtain valuable products [12]. Such upgrading reactions can occur at a single electrode (e.g., CO_2_ reduction at the cathode or oxidative dehydrogenation of alkanes to olefins at the anode) or be coupled between two electrodes. This coupling enables the integration of an endothermic reaction (e.g., cathodic CO_2_ reduction) and an exothermic reaction (e.g., anodic methane oxidative coupling to olefins) in a single device, potentially achieving thermally neutral operation while simultaneously producing valuable products without requiring additional separation steps [8, 13]. In addition, the all‐solid‐state structure enables exceptional stability, and state‐of‐the‐art SOERs for CO_2_ reduction can achieve over 90% Faradaic efficiency and maintain stable operation for more than 1000 h under high current densities [8, 13]. Furthermore, the elevated temperature allows SOERs to operate with superior production efficiency at low overpotentials, benefiting their commercial application.

To date, significant progress has been achieved in SOER development, spanning critical materials innovation, device engineering, and advanced characterization techniques. For instance, advances in electrolyte materials that retain high performance and stability under harsh operating conditions have enabled SOER operation at significantly reduced temperatures, reaching as low as 400°C [14, 15, 16]. Parallel progress has been achieved in the development of electrode materials, particularly oxides capable of forming self‐adaptive surfaces. These include the in situ exsolution of nanoparticles, the strategic surface segregation of active cations, and the generation of oxygen vacancy‐rich surfaces, all of which enhance electrochemical performance and selectively catalyze target reactions [3, 17, 18, 19]. Notably, the application of artificial intelligence (AI) has further accelerated the material discovery and optimization process [20, 21]. Beyond materials development, reaction processes have been intensively investigated through thermodynamic evaluations, kinetic analyses, and numerical modeling to identify optimal operating conditions and maximize product yields [22, 23, 24]. To identify key reaction intermediates and elucidate reaction pathways, advanced characterization techniques have been developed by integrating SOERs with controlled gas supply systems, thermal management units, and electrochemical measurements. For example, synchrotron vacuum ultraviolet photoionization mass spectrometry (SVUV‐PIMS) has been applied to SOER‐based methane coupling reactions, successfully identifying methyl (·CH_3_) radicals as key reactive intermediates in olefin formation, suggesting that ·CH_3_ radicals desorb from the electrode surface and subsequently undergo gas‐phase coupling to form C_2_H_6_, which can further dehydrogenate to C_2_H_4_ [19]. After decades of development, state‐of‐the‐art SOERs can achieve nearly 100% Faradaic efficiency for CO_2_ reduction [13], 90% selectivity for ethane dehydrogenation to ethene [25], and over 70% selectivity toward olefins from propane conversion [26], demonstrating their strong potential for practical chemical upgrading applications [27].

Despite these impressive advances, SOER research has yet to converge toward consistent performance benchmarks or universally applicable design principles, with reported efficiencies and product selectivities often varying widely under seemingly similar conditions. This inconsistency arises from the complex design requirements of critical materials and the challenges in optimizing reaction conditions. Chemical production efficiency is jointly determined by electrochemical efficiency and chemical selectivity. The former is influenced by factors such as the ionic transport number of the electrolyte, electrode activity, electrical conductivity, and diffusion pathways of charge carriers within the electrode. For example, the formation of interconnected proton, electron, and oxygen‐ion‐conducting pathways can significantly enhance electrochemical performance [28, 29]. In contrast, chemical selectivity is primarily governed by the catalytic properties of the electrodes, which are closely related to their defect chemistry and electronic structure. This intrinsic decoupling between electrochemical efficiency and chemical selectivity lies at the heart of many seemingly contradictory SOER reports. For example, P‐SOERs have been reported to exhibit high Faradaic efficiencies for H_2_ production and CO_2_ reduction; however, some studies have yielded low efficiencies of only 40–50% [8, 30, 31, 32]. Similarly, methane oxidative coupling at the anode of O‐SOERs has been shown to produce olefins with high selectivity [33, 34, 35], yet other reports only observe CO and H_2_ formation with no detectable olefins [36]. Nonoxidative dehydrogenation of methane in P‐SOERs has long been hindered by severe carbon deposition, whereas an increase in the operating temperature can induce a transition of the electrolyte from a pure proton conductor to a proton‐oxygen mix conductor, enabling aromatic production while simultaneously suppressing carbon deposition [2]. Such variability originates from the dynamic operating environment of SOERs and the intrinsically sensitive nature of their key components, whose performance is jointly influenced by current density, operating temperature, material composition, and external gas atmospheres. To maximize product yields while maintaining long‐term operational stability, it is essential to systematically summarize the fundamental mechanisms, reaction conditions, and recent advances in the development of critical SOER materials.

**FIGURE 1:**
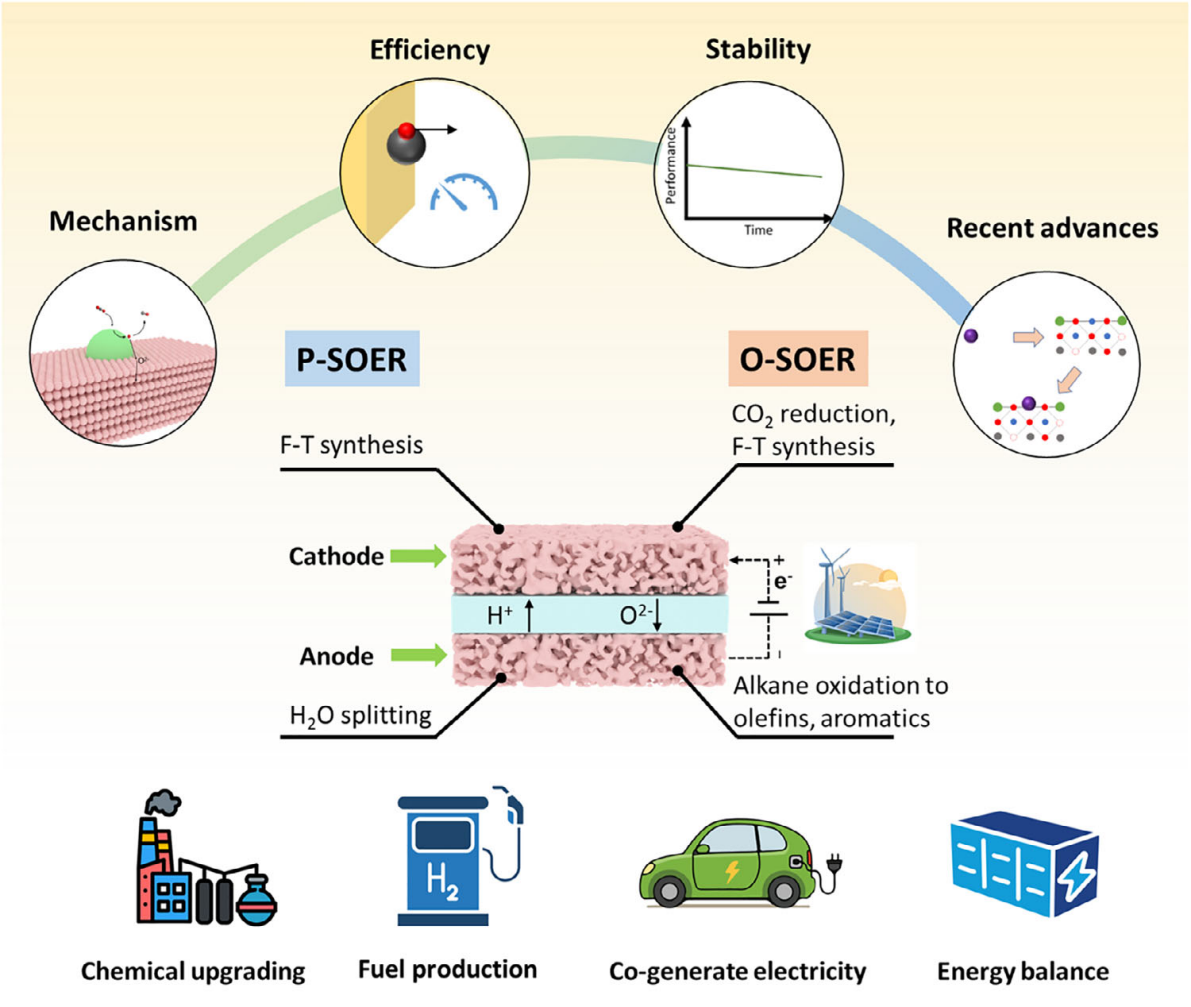
Schematic illustration of SOER and the main contents of this paper.

This article provides a timely and comprehensive review of SOERs, highlighting their broad applications in cathodic CO_2_ reduction and integration with Fischer‐Tropsch (F‐T) synthesis, anodic alkane upgrading, including methane oxidative and nonoxidative coupling to olefins, and alkane dehydrogenation to olefins and aromatics (Figure 1). We first summarize the operating principles of SOERs, the reaction types, and key materials (section 2). Since operating efficiency and long‐term stability are critical determinants of their practical implementation, particular attention is devoted to analyzing the factors governing SOER performance (section 3) and durability (section 4), including electrode and electrolyte material properties, inadequately controlled reaction conditions, carbon deposition, electrode poisoning, and electrode evolution. The cathodic and anodic reactions are then discussed separately, with a comprehensive overview of recent advances in cathode material development (section 5) and the latest progress in anodic valuable product production (section 6). Techno‐economic analyses for chemical production using SOERs are further summarized, highlighting development directions toward reducing application costs. Finally, perspectives and future directions are provided to guide the continued development and eventual commercialization of SOER technologies.

## 2 Reactor types and cell configurations

The SOER types can be classified by their electrolyte, which falls into two categories: oxygen‐conducting and proton‐conducting electrolytes. These electrolytes have opposite charge carrier migration directions, resulting in different reactions at the electrodes. Mixed electron and oxygen ion conductors (MIEC), such as La_1‐x_Sr_x_Co_1‐y_Fe_y_O_3‐δ_ [37], Ba_0.5_Sr_0.5_Co_0.8_Fe_0.2_O_3‐δ_ [38], Sr_2_Fe_1.5_Mo_0.5_O_6‐δ_ [39], and YBa_2_Cu_3_O_6+δ_ [40], can also be used as the membrane in SOERs for alkane partial oxidation and oxygen permeation. This membrane can self‐discharge without needing external electricity contributions, offering advantages of faster reaction kinetics [41, 42, 43]. However, these MIECs generally exhibit poor stability under reducing atmospheres due to the high concentration of transition‐metal cations, limiting their operation in strongly reducing environments. Moreover, their product yields can only be controlled by the chemical potential, which restricts operational flexibility. In contrast, the SOER devices reviewed in this paper consist of two porous electrodes separated by a dense electrolyte layer. Owing to the high ionic transference number of the electrolyte and negligible electronic leakage in the electrolyte. SOERs require external electrical inputs to regulate reaction kinetics. This intrinsic characteristic provides SOERs with an additional degree of control, enabling electrochemical tuning of reaction pathways.

**FIGURE 2:**
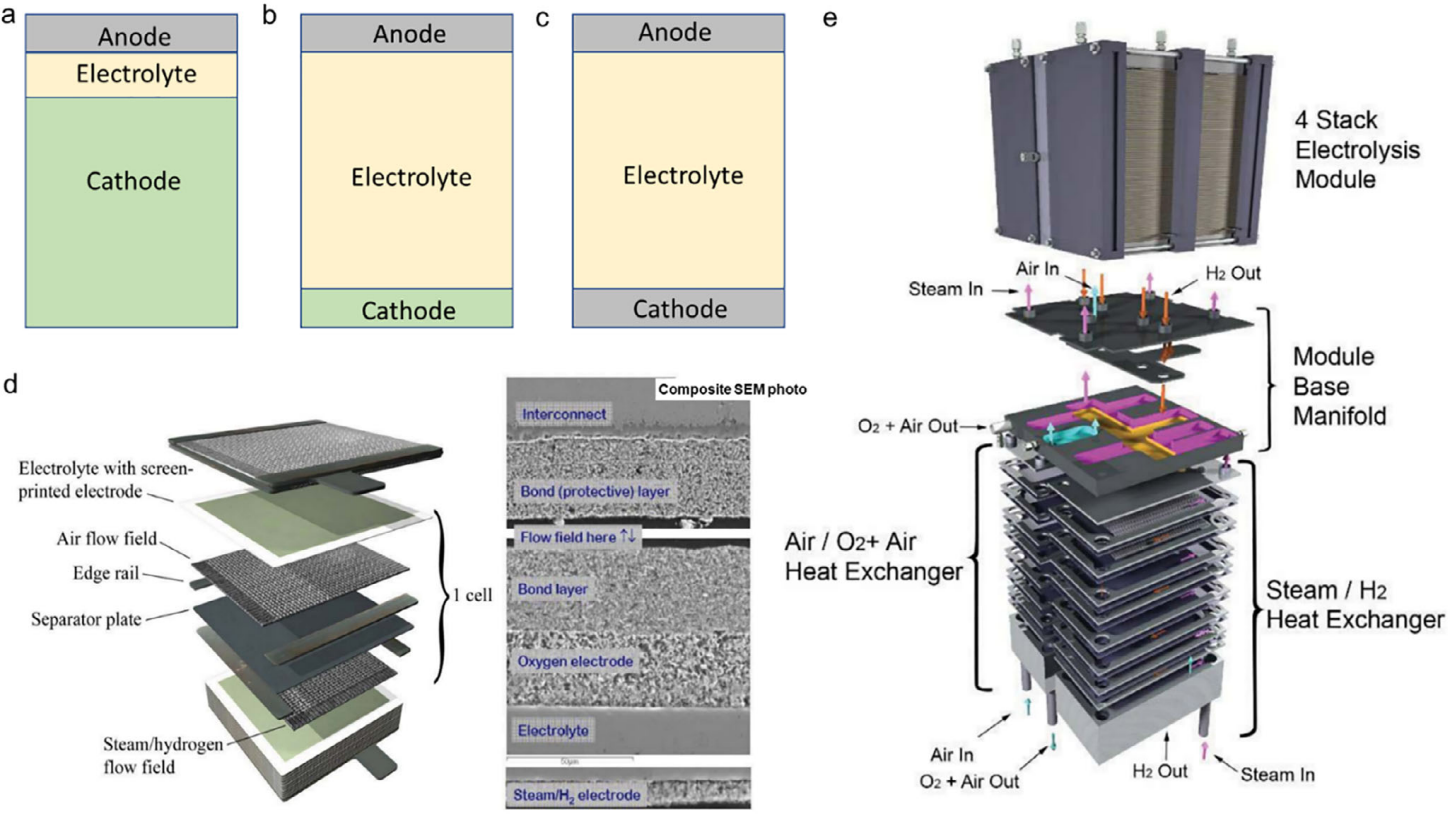
SOER cell configurations: (a) electrode‐supported cell configuration, (b) electrolyte‐supported cell configuration, (c) symmetrical cell configuration. (d) SOER stack construction; the right side shows the scanning electron microscopy. (e) Exploded view of the heat exchanger, base manifold unit, and four‐stack electrolysis unit. Reproduced from reference [59], this material is based upon work supported by the U.S. Department of Energy and is publicly available through OSTI.

In terms of cell configurations, SOERs can be categorized into electrode‐supported (Figure 2a), electrolyte‐supported (Figure 2b), and symmetrical cell configurations (Figure 2c). In electrode‐supported cells, the electrode typically uses nickel‐oxide composites or stainless steel [44, 45], and these electrodes provide the mechanical strength of the device. Furthermore, this configuration enables the deposition of a thin electrolyte layer (<50 µm), resulting in low ohmic resistance and high overall conductivity. However, they are prone to electrode degradation, including nickel diffusion [45], electrode poisoning [46], and carbon deposition [47]. In contrast, electrolyte‐supported cells use a thick electrolyte (typically 300–600 µm) for mechanical support. While the increased thickness leads to higher ohmic losses, this configuration allows greater flexibility in electrode selection, particularly enabling the use of oxide electrodes that combine high activity with improved stability [48, 49, 50, 51], thereby offering flexibility for electrode innovation. Symmetrical cell configurations employ identical electrodes on both sides of the electrolyte and can be achieved in either electrode‐supported or electrolyte‐supported configurations [52]. This architecture simplifies the cell fabrication process, but it requires electrode materials that are redox active and stable, such as Sr_2_Fe_1.5_Mo_0.5_O_6‐δ_ [53], La_0.5_Sr_0.5_Fe_0.9_Nb_0.1_O_3‐δ_ [54], and La_0.75_Sr_0.25_Cr_0.5_Mn_0.5_O_3‐δ_ [55]. For example, a symmetric trilayer architecture of porous YSZ‐La_0.8_Sr_0.2_Cr_0.5_Fe_0.5_O_3‐δ_ (LSCrF) | YSZ | porous YSZ‐LSCrF was fabricated using tape casting followed by hot isostatic pressing. LSCrF is a redox‐stable material capable of operating under both reducing and oxidizing conditions. Postinfiltration of Sm_0.2_Ce_0.8_O_2‐δ_ (SDC) on LSCrF to enhance oxygen‐ion conduction enables the SOER for CO_2_ electrolysis to deliver a current density of 1.3 A cm^−2^ at 1.5 V and 850°C [56].

In addition to the single cell, the SOER stack incorporates current collectors, interconnects, integrating gas‐flow channels, and sealing materials (Figure 2d). Multiple cells are mechanically integrated to form a SOER stack (Figure 2e). Sealing materials can significantly affect gas composition measurements, and commonly used sealing materials include glass sealants, ceramic sealants, and gold rings. Their thermal expansion coefficients and electrical resistivities must be carefully matched with those of the SOER [57]. For glass sealants, the glass transition temperature and melting point should also be considered to match the SOER operating temperatures [58].

### 2.1 Fundamental Operation Principles

**FIGURE 3:**
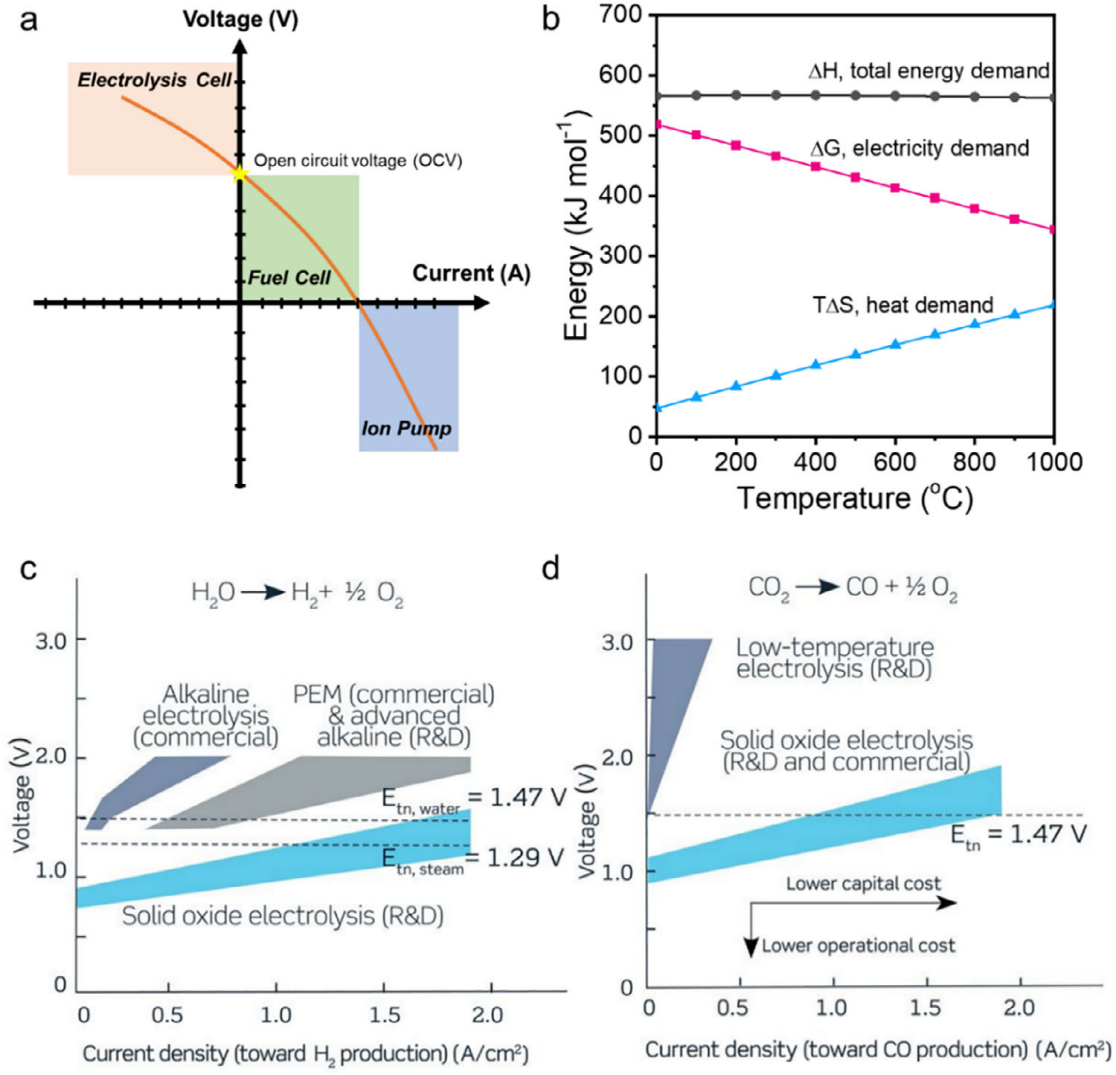
(a) Typical voltage‐current curves for fuel cells, electrolysis cells, and ion pump. (b) Energy‐temperature profiles for Δ*H*, *T*Δ*S*, and Δ*G* for the 2CO_2_(g) = 2CO(g)+O_2_(g) reaction. Typical performance ranges for competing electrolysis technologies for (c) H_2_O splitting and (d) CO_2_ electrolysis. Reproduced with permission [3]. Copyright 2020, the American Association for the Advancement of Science.

The SOER uses either external electricity or the intrinsic chemical potential of the reactants to drive chemical reactions. Its open‐circuit voltage (OCV) is determined by the difference in chemical potential between the anode and cathode (i.e., oxygen partial pressure) according to the Nernst equation. The SOER operates in fuel cell mode when the operating conditions are below the OCV but higher than 0 V. When the applied voltage exceeds the OCV, electrolysis occurs, forcing the reactions at both electrodes to proceed in the reverse direction. In contrast, when the applied voltage is below 0 V, ion pumping occurs, where the external bias drives charge carriers to move from one electrode to the other to align their chemical potential (Figure 3a).

Thermodynamically, the enthalpy change (Δ*H*) of a reaction can be expressed as the sum of a thermal term (*T*Δ*S*) and the Gibbs free energy change (Δ*G*), following the relation Δ*H* = Δ*G* + *T*Δ*S*. In electrochemical systems, Δ*G* corresponds to the maximum reversible electrical work. Taking CO_2_ electrolysis as an example, the total energy demand for the reaction 2CO_2_(g) → 2CO(g) + O_2_(g) remains nearly constant over the temperature range from 0 to 1000°C (Figure 3b). However, as the temperature increases, a larger fraction of the required energy can be supplied by heat (TΔS), thereby reducing the electrical energy demand (ΔG). This explains why high‐temperature SOERs require significantly less electrical input than low‐temperature electrochemical devices (Figure 3c,d). For example, alkaline electrolysis cells and proton exchange membrane (PEM) cells typically require high voltages of over 2 V for water electrolysis and 3 V for CO_2_ reduction, whereas SOERs can perform water electrolysis at voltages as low as 1.3 V and 1.5 V for CO_2_ electrolysis with similar current density [3].

### 2.2 Electrolyte Types

**FIGURE 4:**
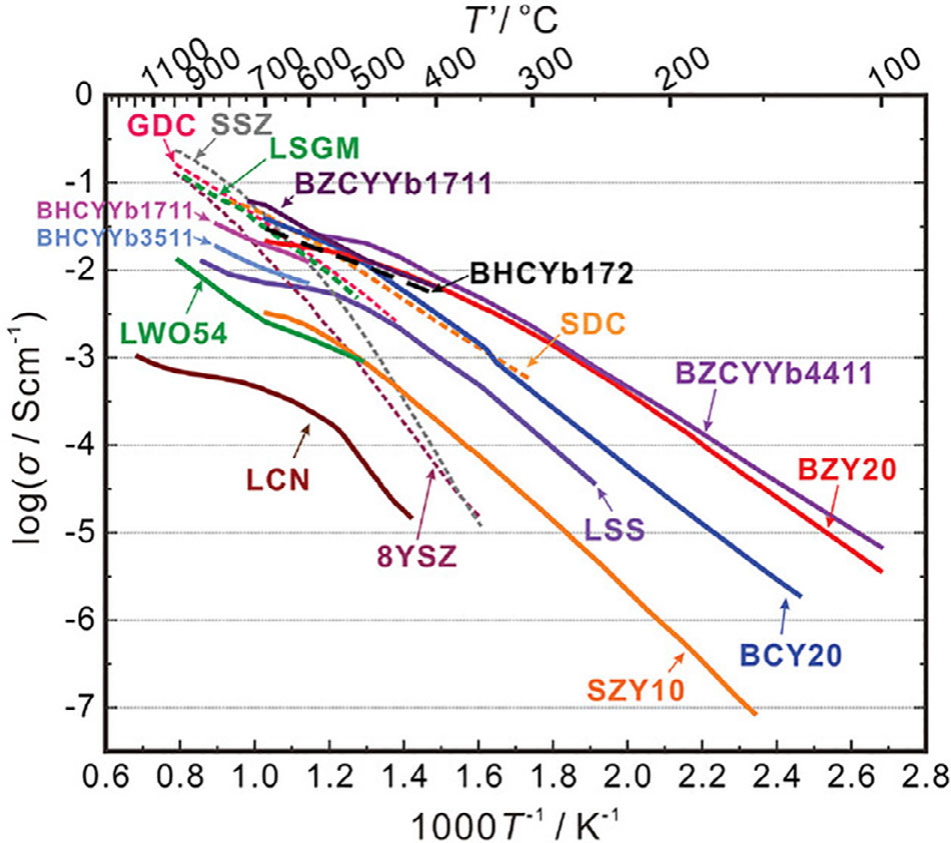
Comparison of conductivity between various electrolytes. Reproduced with permission [71]. Copyright 2024, John Wiley and Sons.

Oxygen‐conducting and proton‐conducting electrolytes are two major electrolyte types in SOERs. Oxygen‐conducting electrolytes, such as yttrium‐stabilized zirconia (YSZ), LaGaO_3_‐based perovskites (e.g., La_0.8_Sr_0.2_Ga_0.8_Mg_0.2_O_3‐δ_), and modified fluorite‐type oxides such as Sm_0.2_Ce_0.8_O_2_, are applicable in membrane reactors. Typically, these electrolytes achieve acceptable conductivity (e.g., >0.01 S·cm^−1^) only at temperatures above 600°C (Figure 4), thus restricting the operation of O‐SOERs to high temperatures to achieve acceptable performance [60]. However, oxygen‐conducting electrolytes typically show a high ion transport number (defined as *t*_ion_ =/) and good stability under wide operation conditions; for example, the oxygen carrier remains the dominant charge carrier (t_ion_ > 0.99) in YSZ in a wide pO_2_ atmosphere from 10^−38^‐0.21 atm [61]. This advantage endows O‐SOER with high Faradaic efficiency in broad operating conditions, typically reaching high Faradaic efficiencies of nearly 100% during water or CO_2_ electrolysis [62]. Furthermore, these electrolytes are less catalytically active due to the minimal number of mobile electrons, which cuts off the charge‐transfer processes between the electrolyte and external reactants, benefiting the chemical stability under harsh conditions [63].

The proton‐conducting electrolyte incorporates proton sources either from water uptake or from hydrogen dissociation. These protons migrate through hopping between adjacent oxygen ions in the BO_6_ octahedron and exhibit higher diffusivity and lower activation energy (0.3–0.4 eV for proton conductors *vs*. >0.6 eV for oxygen ion conductors) [64, 65, 66]. Typical proton‐conducting electrolytes, such as acceptor‐doped BaZrO_3_‐ or BaCeO_3_‐based oxides (e.g., BaZr_0.3_Ce_0.5_Y_0.2_O_3‐δ_ and BaZr_0.1_Ce_0.7_Y_0.1_Yb_0.1_O_3‐δ_), can achieve proton conductivities on the order of 0.01 S·cm^−1^ below 500°C (Figure 4) [15, 67]. This temperature range is well aligned with the operating temperature window of F‐T synthesis reactions, enabling the combination of P‐SOERs with FT synthesis at the electrode. In recent years, heavily scandium‐doped proton conductors have demonstrated exceptionally high proton conductivities [14, 68, 69]. For example, state‐of‐the‐art proton‐conducting electrolytes such as BaSc_0.8_Mo_0.1_W_0.1_O_2.8_ achieve 0.01 S·cm^−1^ at temperatures as low as 193°C [69], opening up possibilities for membrane reactor operation at lower temperatures and suitable for specific chemical reactions, such as liquid chemical production and ammonia synthesis. However, some proton‐conducting electrolytes can undergo cation reduction/oxidation under low/high pO_2_ conditions, forming mobile electrons/holes and leading to a decreased ionic transport number and lower efficiency [30]. Furthermore, proton‐conducting electrolytes typically employ alkaline elements (e.g., Ba, Sr) at the perovskite (ABO_3_) A‐site, which easily react with acidic gases (e.g., CO_2_, SO_2_) and form impurities, making them less stable under harsh conditions [70].

### 2.3 Categories of SOER for Chemical Reactions

**FIGURE 5:**
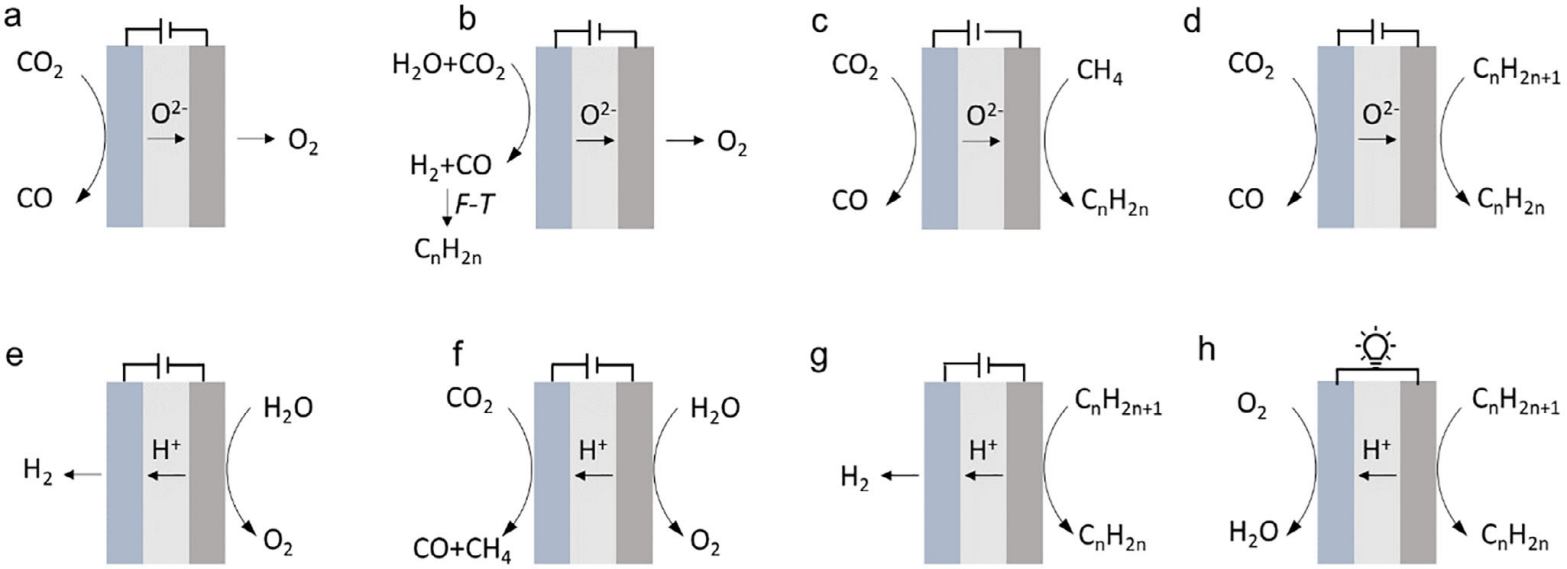
Schematic illustrations of (a) CO_2_ electrolysis in O‐SOER. (b) Combining CO_2_–H_2_O coelectrolysis with F‐T synthesis for producing olefins in O‐SOER. (c) Paring CO_2_ electrolysis and oxidative coupling of methane in O‐SOER. (d) Paring CO_2_ electrolysis and alkane oxidative dehydrogenation in O‐SOER. (e) water electrolysis in P‐SOER. (f) Pooling water electrolysis and CO_2_ reduction in P‐SOER. (g) Alkane nonoxidative dehydrogenation in P‐SOER. (h) Cogeneration of olefins and electricity from alkanes in the P‐SOER.

The SOER can be used for electrolysis (e.g., H_2_O, CO_2_ electrolysis), chemical synthesis (e.g., ammonia synthesis, olefin synthesis), and coupling electrochemical reactions with thermal reactions (e.g., electrolysis combined with F‐T synthesis). Taking CO_2_ electrolysis in O‐SOER as an example (Figure 5a), CO_2_ is directly reduced at the cathode via CO_2_ + 2e^−^ → CO + O^2−^ [3], while the anode undergoes an oxygen evolution reaction (2O^2−^→O_2_+4e^−^). Feeding H_2_O and CO_2_ concurrently in the cathode can trigger coelectrolysis of H_2_O and CO_2_, forming CO‐H_2_ syngas (cathode reaction: H_2_O+CO_2_+4e^−^ → CO +H_2_+ 2O^2−^). Such syngas can further undergo F‐T synthesis when incorporating well‐designed catalysts, producing more valuable products (Figure 5b) [4]. On the anode side, pumped oxygen ions are particularly suitable for oxidizing methane (Figure 5c) or alkanes (Figure 5d) to produce olefins, enhancing their economic attractiveness.

On the other hand, P‐SOERs theoretically cannot directly electrolyze CO_2_ to CO. Alternatively, they can perform steam electrolysis at the anode through 2H_2_O → 4H^+^ + O_2_ + 4e^−^ (Figure 5e), while the cathode reaction proceeds via proton‐promoted CO_2_ hydrogenation: 2H^+^ + CO_2_ + 2e^−^ → CO (CH_4_) + H_2_O (Figure 5f) [5, 6, 7]. The lower operation temperatures in P‐SOERs enable integration with thermal catalysts, making P‐SOERs well suited for upgrading CO_2_ to value‐added hydrocarbons, alcohols, and ethers through thermal catalytic reactions [9]. The P‐SOER can also be used for the nonoxidative dehydrogenation of alkanes (Figure 5g) [25], enabling higher selectivity toward target products. Furthermore, it has been demonstrated to enable the cogeneration of olefins and electricity, wherein the anode undergoes nonoxidative alkane dehydrogenation while the cathode performs the oxygen reduction reaction (Figure 5h) [72].

## 3 Efficiency and Influencing Factors

**FIGURE 6:**
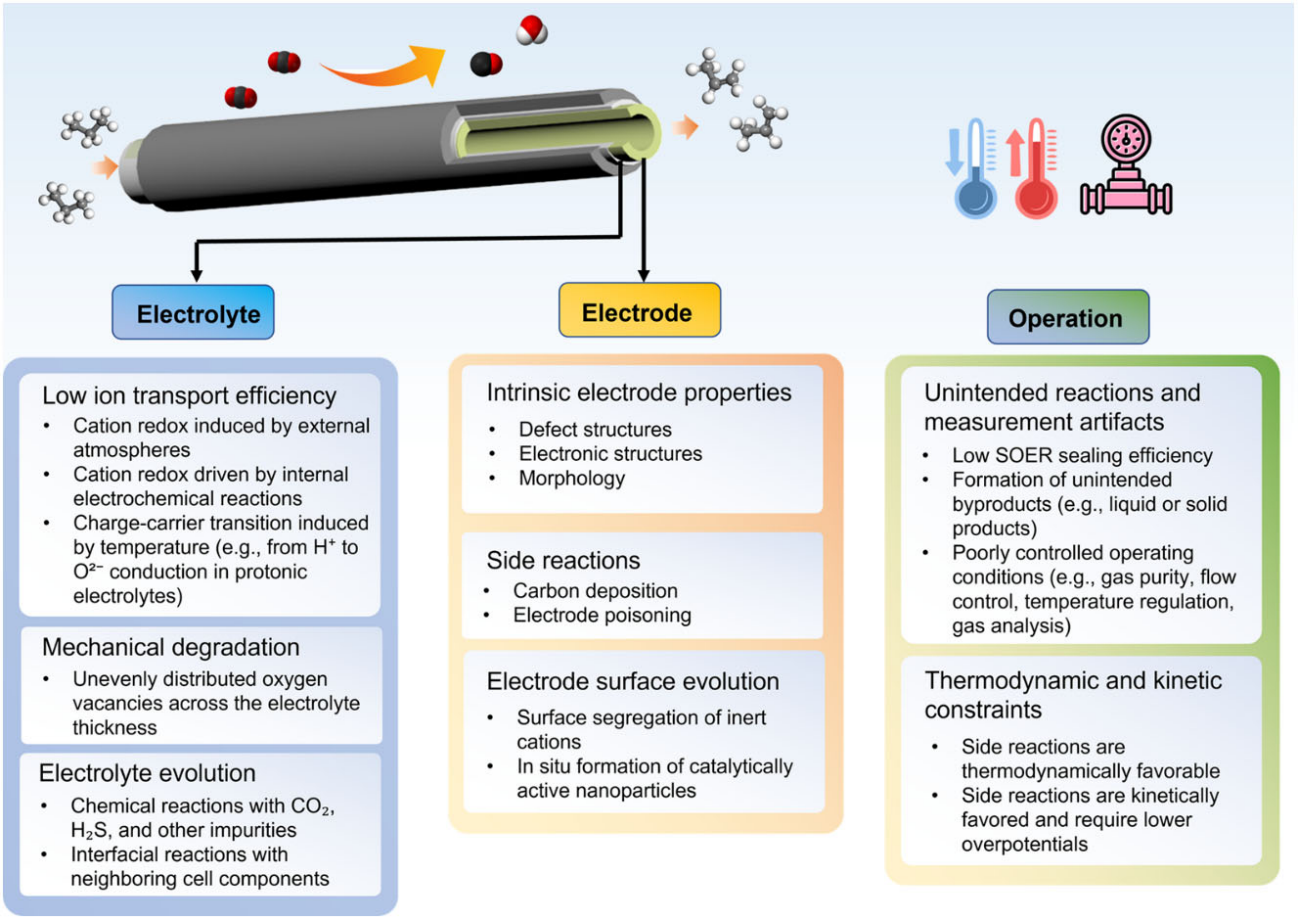
Key factors governing efficiency and stability in SOERs.

Production efficiency is a critical concern in SOERs. It is defined as the ratio of the actual product yield to the total electrons provided (Faradaic Efficiency) [31] or the ratio of product chemical energy to the total energy consumed (Energy Efficiency) [8]. Although many studies have reported impressive electrochemical performance, the actual product yields remain relatively low. Figure 6 and Table 1 summarize the key impact factors that determine the apparent efficiency of SOERs. Overall, the product yield efficiency is governed by both intrinsic material properties and external reaction conditions. In particular, the ionic conductivity of the electrolyte, the catalytic activity of the electrodes, and the extent of competing side reactions all strongly influence the overall yield efficiency.

**TABLE 1:** Factors that affect product efficiency.

Impact components	Impact factors	Support evidence	Proposed mechanisms	Operating conditions	Refs.
Electrolyte	Current density in P‐SOERs	The Faradaic efficiency can be decreased from 90% to ∼40% with increasing electrolysis currents	Higher overpotential leads to high pO_2_ at anode, resulting in hole generation through the reaction of oxygen with oxygen vacancies. The presence of hole decreases Faradaic efficiency	Current density exceeds approximately 0.5A cm^−2^	[23, 30, 32]
	Current density in O‐SOERs	The Faradaic efficiency can increase to nearly 100% with increasing current densities	Low current density results in uncertainty of product detections. Low current density triggers side reactions.	The typical current density is lower than approximately 0.1A cm^−2^	[62, 94, 101]
	Temperature in P‐SOERs	The Faradaic efficiency can be decreased from over 90% below 500°C to ∼50% when approaching 700°C	Higher temperatures favor dehydration reactions, resulting in fewer protons for chemical conversion	The temperature exceeds approximately 650°C	[76, 102]
	Reaction atmosphere in P‐SOERs	The extremely low pO_2_, very high pO_2_, and low pH_2_O usually lead to low Faradaic efficiency	Low pO_2_ promotes cation reduction and electron generation, whereas high pO_2_ favors hole formation. Low pH_2_O leads to an insufficient proton concentration within the electrolyte.	The anode with pure O_2_ or both side with H_2_, or with near dry reaction gas.	[103, 104]
	Sintering adds to the electrolyte in P‐SOERs	Adding sintering decreases Faradaic efficiency	Adding sintering depresses the hydration process and decreases the proton concentration. Formation of impurities at the grain boundary hinders the proton diffusion	Typically, when the sintering add amount exceeds 1 wt. %	[32]
Electrode	Surface reconstruction	Surface reconstruction leads to decreased or increased electrochemical performance/product yields	Form inactive or active secondary phases that hinders/promotes surface catalytic reaction	High temperatures; specific atmospheres (e.g., H_2_O concentrated, CO_2_ contained gases, H_2_‐contained gas)	[105, 106, 107]
	Electrode poisoning	Electrode poisoning leads to electrochemical performance decrease	Specific reaction atmosphere promotes surface poisoning, lattice collapses, and surface reconstruction	High temperatures; the atmosphere contains CO_2_ and SO_2_ gases.	[108, 109]
	Carbon deposition	Carbon deposition results in low or higher selectivity to target products	Carbon deposition covers reaction sites, rupture cells	Under high C/O or C/H conditions. Insufficient oxygen species available. Deep dehydration conditions (e.g., high current densities in P‐SOER)	[106]
Unintended reactions	Impure reactants	Impurities lead to high or low electrochemical performance	The reaction is thermodynamically or kinetically favoring impurity reactions	Low currents	[93]
	Forming liquid products	Liquid products lower the target gaseous products	The untimely removal of liquid products blocks reaction sites. Detection inaccuracy caused by the formation of liquid products	The catalysts favor liquid products; specific working conditions (e.g., low temperatures)	[30]
	Forming hydrocarbons with high carbon atoms	Forming these high hydrocarbons lowers target products, or omitting these products in the calculation leads to inaccuracy in product analysis	These high hydrocarbons are difficult to quantify using GC or MS	Typically, at high current densities, low oxygen/proton‐containing atmospheres	[2]
	Low gas sealing efficiency	Low gas sealing efficiency results in low observed product yields, particularly at low current densities	The products are fast diffused to the counter electrode or atmosphere	Low currents, SOER with small electrode area	[91]

### 3.1 Electrolyte Impacts on Efficiency

Ideally, the electrolyte only permits oxygen ion or proton conduction over a wide range of oxygen partial pressures and temperatures. In oxygen‐conducting electrolytes, oxygen ions migrate through a hopping mechanism between adjacent oxygen sites within the BO_6_ octahedral framework. The overall ionic conductivity is governed by both the oxygen vacancy concentration and the diffusivity of oxygen ions, as described by the Nernst‐Einstein equation: σ = nq^2^D/k_B_T, where σ is the conductivity, n is the mobile charge carrier concentration, D is the diffusion coefficient, k_B_ is the Boltzmann constant, and T is the temperature. However, under electrical bias or at extremely high or low oxygen partial pressures, these electrolytes may exhibit partial electronic conduction, which reduces the overall efficiency of SOERs. Such electronic leakage primarily originates from cation valence changes; for instance, Ce^4+^/Ce^3+^ redox transitions in Sm_0.2_Ce_0.8_O_1.9‐δ_ introduce electronic carriers that hinder efficient ionic transport [73]. Adding an electron‐blocking layer can effectively mitigate this issue. For instance, incorporating a BaCe_0.8_Y_0.2_O_3_ blocking layer between the electrolyte and electrode has been proven to suppress current leakage in ceria‐based electrolytes, increasing OCVs [74].

**FIGURE 7:**
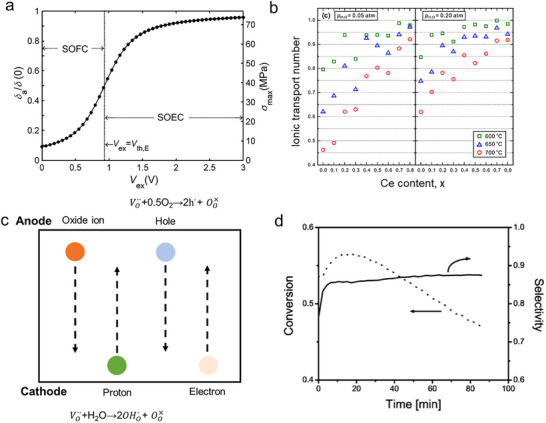
(a) The profile of δ_a_ normalized to δ_(0)_ as a function of V_ex_ in Sm_0.2_Ce_0.8_O_2_ electrolyte under both fuel cell and electrolysis cell conditions, as T = 700°C and *p*O_2_ = 10^−20^ atm. The broken line represents the open‐circuit condition. Reproduced with permission from [75], copyright 2014, Elsevier. (b) Transport number of ionic conduction in BaZr_1‐x_Ce_x_Y_0.2_O_3‐δ_ at 600, 650, and 700°C in wet O_2_ with a pH_2_O of 0.05 and 0.20 atm. Reproduced with permission from [77], copyright 2024, Royal Society of Chemistry. (c) A schematic representation of the existence of partial charge carriers, redrawn from reference [87]. This is an open access article distributed under the terms of the Creative Commons CC BY license. (d) On‐line activity and selectivity data for a 13 wt% chromium‐on‐alumina catalyst plotted against time‐on‐stream for propane dehydrogenation to propene at 550°C. Reproduced with permission [88]. Copyright 2005, Royal Society of Chemistry.

Furthermore, electrochemical reaction‐induced stresses in electrolytes can result in mechanical failure, leading to gas leakage and decreased reaction efficiency. In the Sm_0.2_Ce_0.8_O_1.9‐δ_ electrolyte, chemically induced stresses arise from the inhomogeneous distribution of oxygen nonstoichiometry (δ) across the electrolyte. The oxygen nonstoichiometry equals the vacancy concentration and is a function of the oxygen partial pressure. Numerical calculations were performed to determine the distribution of the normalized quantity δ(x)/δ(0) across the thickness under different operating conditions, reflecting that this uneven distribution of oxygen vacancies can lead to mechanical failure when conducting long‐term operation (Figure 7a) [75].

In P‐SOERs, the electrolyte also encounters challenges related to low efficiency under both reducing and oxidizing atmospheres. Proton‐conducting electrolytes, such as Y‐ or Yb‐doped BaCeO_3_, are susceptible to partial cation reduction under reducing conditions, resulting in the generation of electronic charge carriers and decreases in ionic transference numbers under electrical bias. Moreover, these electrolytes can form electron holes under oxidizing conditions via the oxidation reaction ($O_{2}+V_{O}^{\cdot \cdot }\to 2O_{O}^{\times }+2h^{.}$). This phenomenon is common during steam electrolysis, where the oxygen evolution reaction at the anode oxidizes not only the electrode but also the electrolyte itself. The resulting formation of electron holes further compromises the Faradaic efficiency of the system [76]. Furthermore, at elevated temperatures (e.g., above 700°C), extensive dehydration occurs, leading to reduced proton concentration and lower proton transport number (Figure 7b) [68, 77]. Under these conditions, the chemical potential or the applied electrochemical potential preferentially drives oxygen‐ion conduction rather than proton transport. The coexistence of four charge carriers (protons, oxide ions, electrons, and holes) not only complicates the mechanistic understanding but also leads to reduced Faradaic efficiency and low chemical production efficiency (Figure 7c). For example, the reported steam electrolysis shows that the efficiency can decrease from over 90% at a low current density of 0.1A cm^−2^ to below 50% when the current density exceeds 1 A cm^−2^. This efficiency loss is attributed to enhanced oxidation reactions and Joule heating, which drive a transition from predominantly proton conduction to mixed proton/oxygen‐ion/electron conduction [76]. Carefully designing SOER architectures and optimizing operating conditions can achieve high efficiency; for example, it was found that increasing the thickness of the proton‐conducting electrolyte BaZr_0.1_Ce_0.7_Y_0.1_Yb_0.1_O_3_ from 6 to 10 and 18 µm increases the Faradaic efficiency from 79.05% to 86.56% and 91.53%. This is because thinner electrolytes tend to introduce a higher gradient of concentrations of holes and electrons and Galvani potential, thus introducing more leakage current [78].

### 3.2 Electrode Impacts on Efficiency

The electrode affects the product yield by influencing the elementary reactions, such as adsorption, charge transfer, dissociation, and desorption processes, and promoting the specific elementary reaction can boost the product yields. For example, in the case of cathodic CO_2_ reductions, tailoring the cation deficiency in perovskite (La_0.2_Sr_0.8_)_1‐x_Ti_0.9_Mn_0.1_O_3+δ_ A‐site promotes B‐site cation exsolution and boosts the CO_2_ electrolysis current density by 400%, further enhancing CO production due to the fast oxygen conduction inside the cathode material and the favorable desorption of CO to the gas phase [79]. These in situ exsolution strategies for generating nanoparticles that are triggered by intrinsic properties (e.g., cation deficiency, lattice strain, electrostatic interactions) and external conditions (e.g., electrical stimulation, temperature, and oxygen partial pressure) have been widely applied to SOERs and demonstrated enhanced electrochemical performance together with improved product yields [62, 80, 81, 82, 83, 84]. The enhanced production yield is usually attributed to the finely dispersed nanoparticles, which exhibit enhanced catalytic performance and facilitated charge‐carrier diffusion inside the host oxide, and the strong anchoring effect of these nanoparticles to the host oxide creates tight interfaces that are rich in defects and exhibit optimized electronic structures, leading to high catalytic performance and stability [82, 85].

In addition to the intrinsic properties of the electrode, the electrode evolution, such as poisoning, carbon deposition, and microstructural changes, also significantly affects the SOER efficiency. These processes modify surface characteristics, including the density and nature of active sites, defect chemistry, and electronic structures. For example, carbon deposition on metal‐oxide catalysts is frequently observed to decrease the overall conversion for propane dehydrogenation, yet it can simultaneously enhance or maintain the selectivity toward olefins (Figure 7d) because the formed carbon covers the active sites, hindering the cleavage of the C‐C bond and thus maintaining the selectivity toward C_3_H_6_ [86].

### 3.3 Unintended Reactions and Thermodynamic Constraints

**FIGURE 8:**
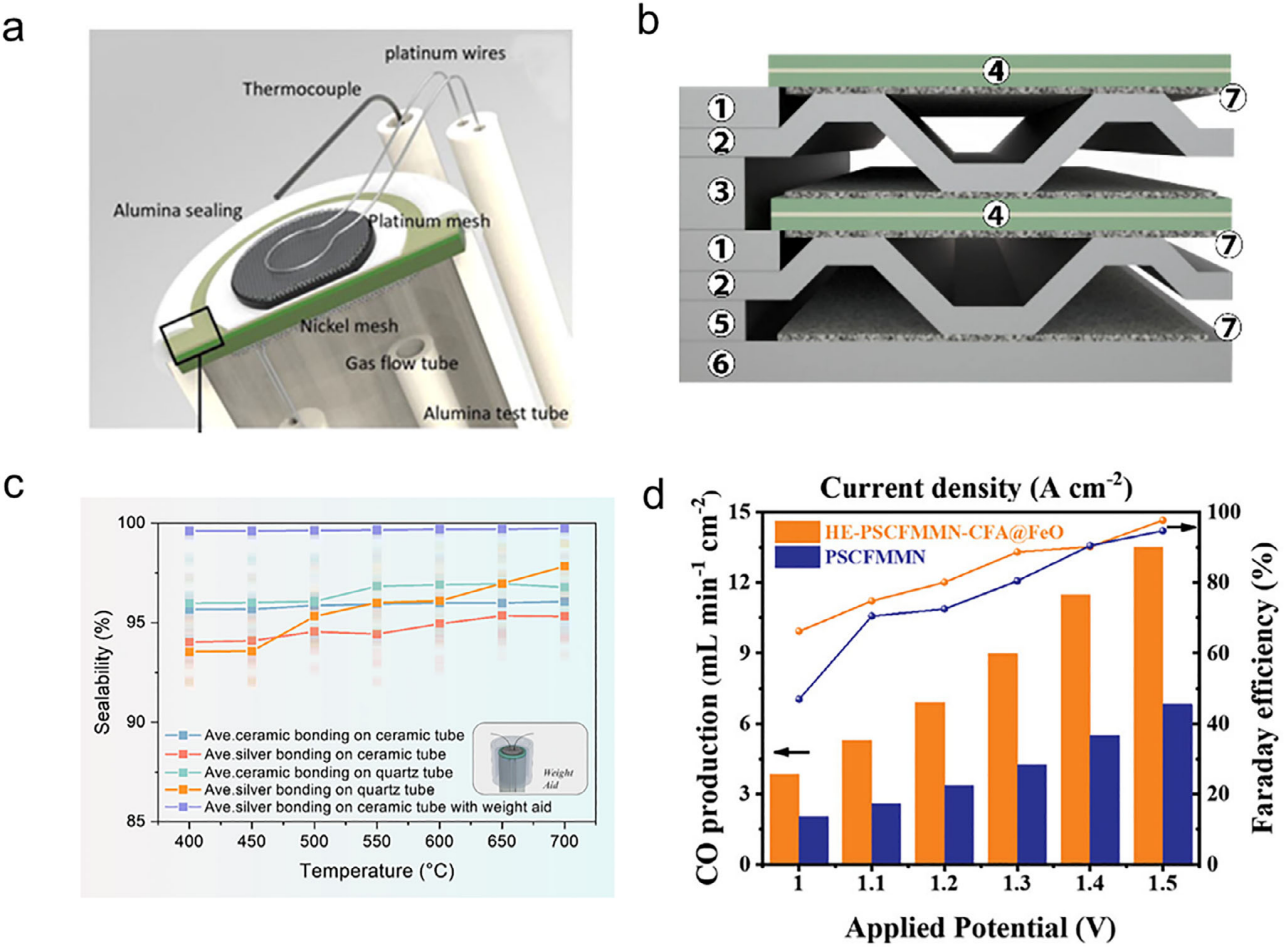
(a) Schematic illustration of the electrode‐supported cell test setup. Reproduced from reference [89] under the terms of the Creative Commons Attribution License (CC BY). (b) Large‐sized, planar SOER unit, with numbers referring to 1: Oxygen spacer; 2: interconnect; 3: hydrogen spacer; 4: symmetrical cell; 5: auxiliary spacer; 6: bottom current collector; 7: contact foam. Reproduced from reference [90] under a Creative Commons Attribution 4.0 International License. (c) Comparison of the average sealabilities of five sealing strategies based on the same testing conditions. Reproduced with permission [91]. Copyright 2024, Royal Society of Chemistry. (d) Faraday efficiency of the SOERs with different cathodes (Pr_0.4_Sr_0.6_Cu_0.3_Fe_0.4_Mo_0.1_Mn_0.1_Nb_0.1_O_3‐δ_ or Pr_0.8_Sr_1.2_(CuFe)_0.4_Mo_0.2_Mn_0.2_Nb_0.2_O_4‐δ_‐CuFe alloy@FeOx) in pure CO_2_ at applied voltages from 1.0 to 1.5 V at 800°C. Reproduced with permission [94]. Copyright 2023, John Wiley and Sons.

Unintended reactions occurring during SOER operation are another factor influencing the observed product yields. Such reactions may arise from insufficient experimental control. In terms of product analysis in SOER devices, one of the primary challenges lies in maintaining gas‐tight sealing to prevent oxygen intrusion from ambient air. In laboratory‐scale button cells, alumina‐based or glass sealants are typically employed to ensure gas tightness (Figure 8a) [89]. In contrast, for large‐area planar SOERs (Figure 8b), glass sealants combined with applied mechanical pressure are more widely used to achieve reliable sealing [90]. However, these sealants often exhibit porous microstructures or require specific operating temperatures to maintain effective sealing. It has been observed that different sealants applied to alumina‐ or quartz‐based test holders exhibit different sealing efficiencies. These sealing efficiencies can be further improved by applying an external weight to the SOER device; however, in some cases, the sealing efficiency is below 95% (Figure 8c) [91]. Such insufficient sealing is particularly detrimental for small‐sized SOERs; for example, a high current density of 1 A cm^−2^ during CO_2_ electrolysis corresponds to a change of approximately 3% in the total outlet gas composition when the inlet gas flow rate is 100 sccm, assuming an effective electrode area of 0.5 cm^2^ and a Faradaic efficiency of 100%. Under such conditions, even a small gas leak arising from imperfect sealing can substantially affect gas composition measurements. Furthermore, although the electrochemical performance remains stable during testing, fluctuations in the outlet gas composition are often observed, proving that evaluating SOER performance solely based on electrochemical metrics does not fully reflect its true operational behavior [76].

In addition to the sealing efficiency, the impurities inside the reaction gas also lead to inaccuracies in the product determinations; for example, in CO_2_ electrolysis in O‐SOERs, high‐purity CO_2_ is required to avoid unintentional oxygen pumping before CO_2_ electrolysis initiates. Because the oxygen‐pumping reaction is both thermodynamically and kinetically more favorable than CO_2_ reduction in SOERs [92], this leads to deceptively high current densities but very low Faradaic efficiencies. This phenomenon explains why high Faradaic efficiencies in CO_2_ electrolysis are often observed only at high current densities because oxygen pumping occurs prior to CO_2_ reduction, particularly at low currents (Figure 8d) [62, 93, 94].

In addition, the formation of unaccounted products, such as liquid‐phase species or higher‐carbon‐number hydrocarbons in SOERs, can further reduce the apparent product efficiency if these products are not properly quantified. These products are difficult to detect by gas chromatography and are often poorly quantified by mass spectrometry; excluding these species from quantification inevitably leads to underestimated efficiencies. For example, in P‐SOER‐driven CO_2_ reduction coupled with F‐T synthesis, methanol is formed [30]. Although minor, omitting methanol from the analysis lowers the apparent efficiency. Similarly, hydrocarbons with high carbon numbers may form during carbon deposition, and excluding these products also lowers the apparent efficiency [95].

Kinetically or thermodynamically favorable side reactions can compete with the desired reactions. For instance, the reverse water‐gas shift reaction (CO_2_ + H_2_ → CO + H_2_O) is thermodynamically favored at temperatures above 600°C, whereas methanation is favored at lower temperatures. As a result, CO_2_‐H_2_O coelectrolysis in O‐SOERs operating at high temperatures typically produces CO‐rich syngas, while P‐SOERs often yield products with higher CH_4_ content [92]. In the case of ammonia synthesis using P‐SOERs, the cathode favors the hydrogen evolution reaction over nitrogen reduction, leading to Faradaic efficiencies below 10% in many reported studies [96, 97]. Overcoming these competing pathways requires first exploiting thermodynamic or kinetic factors that favor the target reactions, followed by the design of catalysts capable of selectively promoting the desired pathways.

In addition to tailoring the operating temperature and reactant composition, the solid‐state nature of SOERs also enables operation under elevated pressures, which can enhance production efficiency and render specific reactions more thermodynamically favorable. Numerical studies conducted on a 90‐cell SOER stack predict higher open‐circuit voltages, lower area‐specific resistance, and reduced pressure drop across the flow fields at elevated operating pressures [98]. Experimental studies have also been performed on Ni‐YSZ|YSZ|La_0.6_Sr_0.4_Co_0.2_Fe_0.8_O_3_‐gadolinium‐doped Ceria SOERs at 800°C for the coelectrolysis of CO_2_ and steam. The results show that pressurized coelectrolysis shifts the limiting current density to higher values. Although the OCV increases under 10 bars, the overall cell performance is significantly improved at 1.3 V under pressurized conditions. Furthermore, methane formation was observed only at 10 bar [99]. Similar experiments have been conducted using a tubular SOER for syngas production from CO_2_ and steam over a pressure range of 1–8 bar. In fuel cell mode, the device exhibited a 44.2% increase in maximum power density, while in electrolysis mode, performance was also enhanced at the same applied voltage. These improvements are attributed to reduced polarization resistance and increased OCV [100]. However, operation under elevated pressures imposes more stringent requirements on sealing materials and experimental setups, addressing the importance of developing more efficient sealing materials.

## 4 Stability and Influencing Factors

Stability is another critical factor influencing the practical application of SOERs. In SOERs used for upgrading carbon‐containing chemicals, carbon deposition, electrode poisoning, electrode evolution at elevated temperatures, and electrolyte instability under harsh operating conditions are the primary factors governing SOER stability.

### 4.1 Carbon Deposition

**FIGURE 9:**
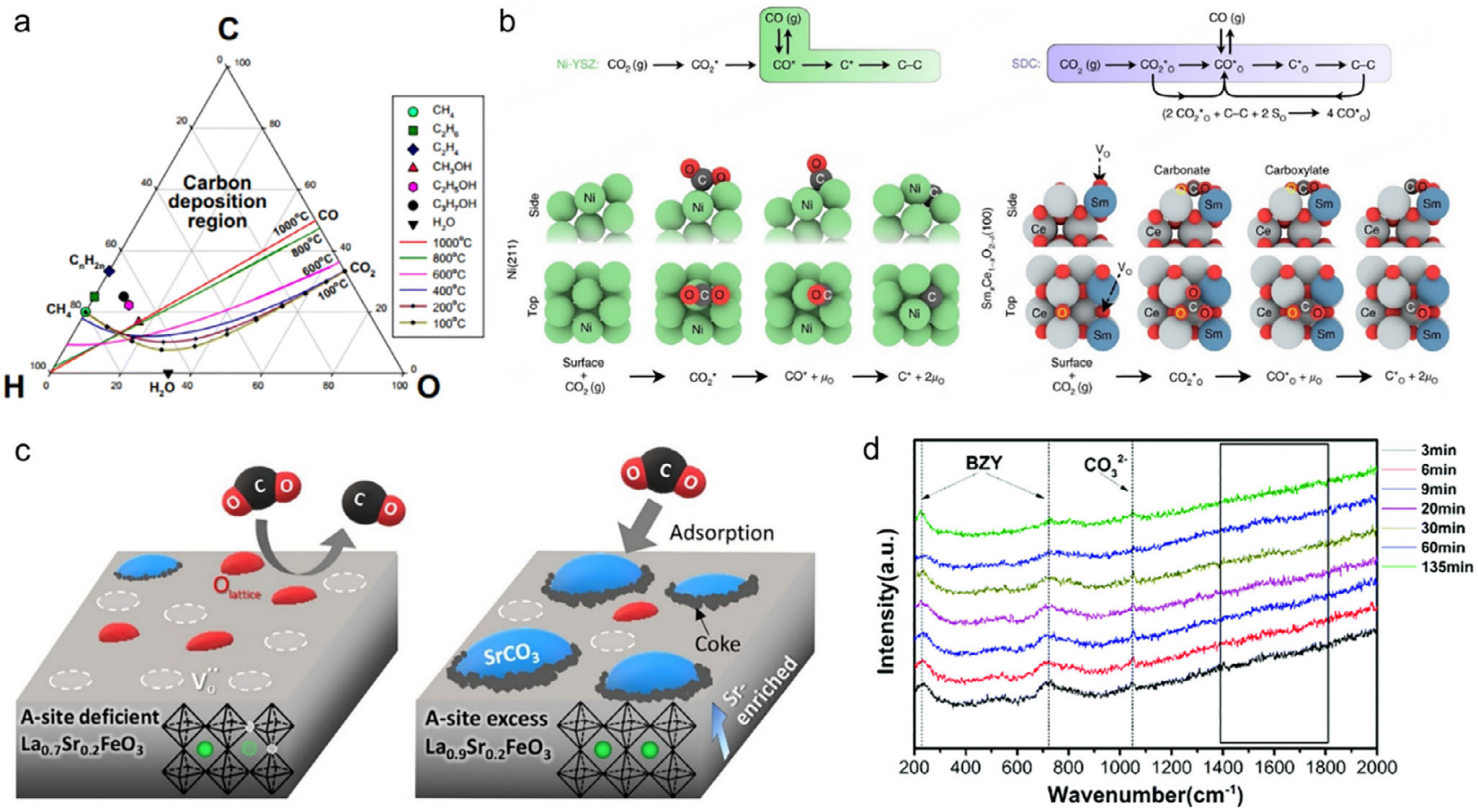
(a) Phase diagram of C‐H‐O to show thermodynamic carbon deposition under various temperatures. Reproduced with permission [140]. copyright 2003, IOP Publishing. (b) Reaction pathways of CO_2_ reduction over Ni‐YSZ and SDC. Reproduced with permission [120]. Copyright 2019, Springer Nature. (c) Schematic illustration of SrCO_3_ formation hindering CO_2_ adsorption. Reproduced with permission [106]. Copyright 2021, Elsevier. (d) Raman spectrum evolution of the Ni‐BaZr_0.8_Y_0.2_O_3‐δ_ sample measured at 550°C in 6% CO_2%_–24% H_2%_–70% Ar. It was shown that CO_3_^2−^ species were formed during CO_2_ reduction. Reproduced with permission [30]. Copyright 2019, Royal Society of Chemistry.

Carbon deposition is a common issue when the electrode is exposed to hydrocarbon fuels or when carbon‐containing products (e.g., CO) are generated. Thermodynamically, carbon formation is governed by the carbon‒oxygen‒hydrogen ratio and operating temperature. Higher C/O or C/H ratios lead to a greater tendency for carbon formation (Figure 9a) [95, 110, 111, 112]. Typically, carbon deposition follows a nucleation‐growth mechanism, in which acid sites usually serve as active centers for hydrocarbon cracking and carbon atom formation. These carbon species can subsequently migrate, aggregate, and grow into carbon filaments or films that cover catalytic sites and block reaction pathways [85, 113]. Excessive carbon growth may exert mechanical stress on the electrode and electrolyte, causing SOER rupture and rapid degradation in electrochemical performance. For instance, CO_2_ electrolysis in SOERs frequently encounters carbon deposition due to the CO disproportionation reaction (2CO → CO_2_ + C), which is more likely to occur at lower temperatures, high current densities, and low electrode porosity [114].

Mitigating carbon deposition to prolong the SOER lifetime can be achieved either by optimizing reaction conditions to operate outside the carbon‐deposition region or by designing catalysts capable of enhanced oxygen/proton storage. For example, coelectrolysis of CO_2_ and H_2_O may enhance stability by supplying excess H and O into the system, thereby preventing carbon accumulation [115, 116, 117]. On the other hand, introducing proton‐conducting oxides, such as BaZr_0.3_Ce_0.5_Y_0.2_O_3‐δ_ and BaZr_0.1_Ce_0.7_Y_0.1_Yb_0.1_O_3‐δ_, these proton‐conducting oxides are highly hydrophilic and can generate a water‐rich surface layer, which is beneficial for mitigating carbon deposition by promoting carbon oxidation [118]. Similarly, the application of oxygen‐rich catalysts, such as CeO_2_‐based materials, enables stable long‐term operation even in an atmosphere enriched with methane. For example, Ce_0.9_Ni_0.05_Ru_0.05_O_2_ catalysts incorporated into a Ni‐ BaZr_0.1_Ce_0.7_Y_0.1_Yb_0.1_O_3‐δ_ anode achieved stable operation for ∼550 h at 500°C using nearly dry methane as fuel [119]. It was also shown that carbon atoms on the surface of CeO_2_‐based materials, such as Sm_0.2_Ce_0.8_O_2‐δ_ and Ni–Sm_0.2_Ce_0.8_O_2‐δ_ can be trapped as carbon oxide intermediates, altering the reaction pathway and mitigating carbon deposition during CO_2_ electrolysis (Figure 9b) [120]. In recent years, it was found that carbon deposition can be suppressed by developing catalysts that are able to grow nanoparticles in situ on oxide surfaces. These in situ exsolved nanoparticles have robust interfacial bonding to the host oxide, thus effectively preventing particle detachment by carbon growth and enhancing catalytic stability in hydrocarbon‐containing environments [72, 85].

In addition to catalyst development, carefully controlling the reaction conditions can promote carbon deposition. The O‐SOER anode naturally generates oxygen species to partially oxidize the carbon deposition through pumping oxygen; for example, the continuous pumping of oxygen to oxidize anodic CH_4_ in O‐SOER can largely suppress carbon deposition at the CH_4_ side [121]. Soft carbon can be removed through the injection of more hydrogen into the reaction chamber; however, in some practices, hard carbon consisting of a highly cross‐linked, amorphous carbon network with mixed sp^2^/sp^3^ hybridization will be formed on the catalyst after long‐term testing, and the removal of hard carbon typically requires the involvement of combustion treatments [122]. Unfortunately, this combustion process can rapidly oxidize the electrode materials, particularly Ni‐oxide ceramic electrodes, which results in severe thermal shock and rupture of the cell.

### 4.2 Electrode Poisoning

Electrode poisoning results from the undesirable adsorption of reaction intermediate species or external species. Such adsorption can cover the reaction site or even react with catalysts to form impurities, alter the surface properties, and hinder further reactions. The sulfur poisoning in hydrocarbon synthesis and Cr poisoning from connectors are representative of electrode poisoning [123, 124, 125]. The investigation results have shown that ppb‐level sulfur impurities in the reaction gas can result in resistance increases for CO_2_ electrolysis, forming sulfur‐containing species in electrodes. Thermodynamic calculations indicate that the nickel‐based electrodes easily encounter sulfur poisoning by forming Ni_3_S_2_ in hydrogen‐poor regions, and this solid Ni_3_S_2_ phase becomes less stable and transforms into a liquid phase at high temperatures. In contrast, NiSO_4_ forms in oxygen‐rich environments when reacting with O_2_, CO_2_, and H_2_O [125]. In addition to sulfur and Cr, P can form Ni_5_P_2_ and NiP_3_, while B can form Ni_4_B_3_ and NiB, and these phases remain stable even at temperatures up to 800°C. The electrode can also react with Cl_2_ to form NiCl_2_, which remains stable even at Cl_2_ concentrations as low as 100 ppb [125].

Altering the reaction conditions or developing anti‐poisoning catalysts can enhance electrode stability. For example, it was found that adding hydrogen into the reaction chamber can suppress such sulfur poisoning [126]. From the materials aspect, infiltrating catalysts with enhanced anti‐sulfur poisoning proves feasible. For example, infiltrating cobalt into Ni‐Gd_0.1_Ce_0.9_O_2_ can largely inhibit such poisoning [127]. In addition to catalyst modification, the utilization of a versatile SOER electrode surface can prolong the lifetime of the SOER. For example, SrCrO_4_ can be formed over the La_0.6_Sr_0.4_Co_0.2_Fe_0.8_O_3‐δ_ surface after treating the sample in the presence of Fe‐Cr interconnects at 800°C for 40 h; however, after a prolonged treatment time, LaCrO_3_ emerged, and the electrochemical performance gradually increased due to the much higher electrical conductivity of LaCrO_3_ compared to SrCrO_3_ (3.4 × 10^−1^ S cm^−1^ vs. 1.8 × 10^−4^ S cm^−1^). Inspired by this, the transition from SrCrO_4_ to LaCrO_3_ can be deliberately promoted by switching the operating mode to electrolysis mode because the higher oxygen partial pressure at the air electrode promotes SrCrO_4_ thermodynamic transformation into LaCrO_3_ [123].

### 4.3 Electrode Evolution

Electrode evolution is frequently encountered in SOERs and other high‐temperature devices due to the versatile nature of electrode materials at high temperatures [79, 128, 129]. This surface reconstruction is influenced by the combination of external conditions (e.g., temperature, atmosphere, electrical bias) and internal material properties (e.g., lattice strain, cation deficiency, electrostatics) [130, 131]. Such surface reconstruction can lead to positive electrochemical performance enhancements or negative performance degradation depending on the nature of the newly formed secondary phase [80, 82, 132, 133].

Inert cation surface segregation, such as barium and strontium, is frequently observed and usually leads to performance degradation. The segregated cations can react with external reactants to form crystalline phases or act as intermediates in electrochemical reactions. During CO_2_ electrolysis, surface segregation of inert cations is frequently observed because acidic CO_2_ reacts with alkaline A‐site cations in perovskite oxides. Even when these perovskites function as counter electrodes and are not directly exposed to CO_2_, trace amounts of CO_2_ leaking from the cathode to the anode, as well as residual CO_2_ in the ambient atmosphere, can induce cation surface segregation and surface reconstruction. For example, SrCO_3_ is formed over the La_0.7_Sr_0.2_FeO_3_ surface during CO_2_ electrolysis, and the stronger adsorbed carbonate can lead to a complete reduction of CO_2_ during electrolysis to form carbon, hindering further reactions (Figure 9c) [106]. In some cases, carbonate species act as intermediates during CO_2_ reduction and do not necessarily form crystalline carbonate impurities, particularly under conditions that can rapidly remove carbon‐containing species. For example, CO_3_^2−^ species have been identified on the BaZr_0.8_Y_0.2_O_3‐δ_ surface during CO_2_ reduction in P‐SOERs based on Raman spectroscopy; however, high‐resolution TEM reveals that no carbonate secondary phase is formed (Figure 9d) [30]. Furthermore, such surface evolution can be modulated by altering the reaction mode, as changes in chemical potential modify lattice distortion and surface defect structures. For example, a recent study reported that Sr segregation is inevitable during CO_2_ electrolysis on La_0.6_Sr_0.4_Co_0.2_Fe_0.8_O_3‐δ_ air electrodes, yet anodic polarization was found to effectively suppress this segregation [134]. Electrode surface reconstruction can also enhance electrochemical performance under specific conditions. Such reconstructions modify surface defect states and electronic structures or lead to the formation of catalytically active secondary phases. A widely adopted strategy involves triggering nanoparticle exsolution by reducing electrode materials under hydrogen atmospheres or by applying an electrical bias [135, 136, 137]. Exsolved nanoparticles, such as nickel, can significantly facilitate CO_2_ dissociation in SOERs and promote alkane decomposition reactions [62, 138].

Beyond intrinsic electrode property evolution, the electrode microstructure can also undergo significant changes during operation. This effect is particularly pronounced in nickel‐based electrodes, where nickel diffusion can occur near the electrode‐electrolyte interface. For example, the influence of operating temperature on the durability of SOECs under galvanostatic conditions has been investigated using EIS combined with postoperative microstructural analysis. The stability tests revealed a higher degradation rate at lower operating temperatures. SEM characterizations of the degraded cell showed that the porosity in the active electrode region (within ∼7 µm of the electrode‐electrolyte interface) was increased due to nickel diffusion. This increase in porosity reduces the density of active sites, thereby decreasing the overall energy efficiency of the cell [139].

### 4.4 Instability of Electrolytes

The electrolyte can be the bottleneck to the stability of SOERs. This stability issue may arise either from the electrolyte‐electrode interface reaction or from the electrolyte itself degradation. La_1‐x_Sr_x_Ga_1‐y_Mg_y_O_3‐δ_ (LSGM) has been proven suitable for low‐temperature O‐SOERs due to its high conductivity compared to ZrO_2_‐based electrolytes; however, LSGM frequently encounters interfacial problems when cosintered with nickel‐based electrodes or other perovskites. It has been found that when cosintered with Ni‐Gd_0.2_Ce_0.8_O_2‐δ_, the formation of a La‐deficient La‐Sr‐Ga‐O phase at the LSGM/NiO or LSGM/CeO_2_ interface can hinder the electrochemical performance due to the highly resistive nature of this phase under operating conditions [141]. In addition to reacting with nickel, such electrolytes can also react with perovskites, such as La_0.6_Sr_0.4_Co_0.2_Fe_0.8_O_3‐δ_, and after cosintering at 1100°C, high‐resistance phases form and decrease the electrochemical performance. The ToF‐SIMS results confirm the bulk diffusivities of Co and Fe in LSGM [142].

In addition to the interface problem, the electrolyte itself can encounter stability problems in harsh conditions, particularly in proton‐conducting electrolytes in P‐SOERs when operating under acid gas atmospheres. The proton‐conducting electrolytes typically use alkaline elements in the A‐site, such as Ba or Sr; however, this can react with acid gas such as CO_2_ and H_2_S to form BaCO_3_ or sulfates under elevated temperatures. For example, to enhance the sulfur tolerance for proton‐conducting electrolytes, BaZr_0.1_Ce_0.7_Y_0.1_Yb_0.1_O_3_ was developed and successfully operated in hydrocarbon fuels containing H_2_S [15]; however, it remains unstable under CO_2_ atmospheres [70]. It was found that increasing the Zr content and formulating BaZr_0.4_Ce_0.4_Y_0.1_Yb_0.1_O_3_ showed much enhanced stability both in H_2_O and CO_2_ atmospheres [143, 144], and this phenomenon was also observed in BaZr_x_Ce_0.8‐x_Y_0.2_O_3‐δ_ electrolytes [145, 146]. Kinetic analysis of the isothermal solid‒gas reaction indicated that these electrolytes were partially carbonated according to a first‐order nucleation/nuclei growth mechanism, in which oxygen vacancies act as active nucleation sites [147]. In addition to its reactivity with acidic gases, BZCYYb also exhibits considerable chemical reactivity toward alumina, zirconia, and cerium oxide substrates during sintering. Such interactions can induce significant Ba volatilization and even cause the perovskite phase to decompose, necessitating strict control over processing conditions to prevent these detrimental reactions [148].

## 5 SOER for Cathodic CO_2_ Reduction

CO_2_ reduction using SOERs has emerged as a rapidly growing research topic in recent years, owing to its high efficiency and operational robustness. In O‐SOERs, CO_2_ reduction proceeds at the cathode via direct electrochemical reactions, whereas in P‐SOERs, CO_2_ reacts with electrochemically supplied protons at the cathode through thermocatalytic pathways.

### 5.1 Reaction Mechanisms and Critical Requirements for Cathodes

In O‐SOERs, CO_2_ first undergoes adsorption, followed by a series of charge‐transfer steps and subsequent CO_2_ dissociation to form CO and surface oxygen species. These oxygen species migrate through the hopping between oxygen vacancies in the cathode, migrate to the oxygen‐conducting electrolyte, and then release oxygen in the anode. Many studies have identified carbonate species as the key intermediates during CO_2_ activation and dissociation processes in ZnO and CeO_2_ catalysts, which is confirmed by Raman characterization results and DFT calculation results [149, 150, 151, 152]. The computational calculations on La_0.75_Sr_0.25_FeO_3‐δ_ catalysts verified that the CO_2_ reduction process follows Equations ((1), (2), (3), (4)). In the whole CO_2_ reduction process, CO_2_ can react with lattice oxygen to form carbonate species, with the C═O bond bending due to the charge transfer between CO_2_ and the catalyst [152].

(1) $$CO_{2}\left(\mathrm{gas},\mathrm{liner}\right)+O^{2-}\left(\mathrm{lattice}\right)\to {\mathrm{CO}}_{3}^{2-}\left(\mathrm{ad}.\right)$$

(2) $${\mathrm{CO}}_{3}^{2-}\left(\mathrm{ad}.\right)+e^{-}\to {\mathrm{CO}}_{2}^{-}\left(\mathrm{activated},\mathrm{bent}\right)+O^{2-}\left(\mathrm{lattice}\right)$$

(3) $${\mathrm{CO}}_{2}^{-}\left(\mathrm{activated},\mathrm{bent}\right)+e^{-}\to \mathrm{CO}\left(\mathrm{ad}.\right)+O^{2-}\left(\mathrm{ad}.\right)$$

(4) $$\mathrm{CO}\left(\mathrm{ad}.\right)\to \mathrm{CO}\left(\mathrm{gas}\right)$$

In P‐SOECs, CO_2_ reduction proceeds through thermocatalytic reactions between CO_2_ and protons [8]. These proton species are generated via steam electrolysis at the anode and subsequently transported to the cathode (Equation 5), where they react with CO_2_ to produce CO or hydrocarbons (Equation 6). A unique advantage of P‐SOECs is their inherently lower operating temperature compared with O‐SOECs, enabling more thermodynamically favored F‐T synthesis under intermediate‐temperature conditions. Previous studies have demonstrated that lower temperatures (e.g., ∼450°C) promote CH_4_ formation, whereas higher temperatures promote CO production [30]. However, compared with O‐SOERs, P‐SOERs generally exhibit lower efficiency due to undesirable electronic (hole/electron) conduction in proton‐conducting electrolytes and the stringent requirements for catalysts with high selectivity.

(5) $$2H_{2}O\to 4H^{+}+O_{2}+4e^{-}$$

(6) $$2H^{+}+CO_{2}+2e^{-}\to \mathrm{CO}+H_{2}O$$

The cathode for CO_2_ reduction in SOERs should possess high catalytic activity, good mixed conductivity, including electron/oxygen ion conductivity for O‐SOERs and electron/proton conductivity for P‐SOERs, and excellent chemical stability under harsh operating environments [9, 153, 154]. Initially, metal catalysts were employed; subsequently, oxides and metal‐oxide heterogeneous catalysts were developed to enhance catalytic performance while reducing costs.

### 5.2 Recent Advances in Cathode Materials for CO_2_ Electrolysis

#### 5.2.1 Metal‐Based Electrodes

Early studies primarily employed metal electrodes or nickel‐oxide composite cathodes for CO_2_ reduction because of their high electronic conductivity and promising catalytic activity for CO_2_ dissociation. However, these electrodes typically require the introduction of a protective reducing gas (e.g., H_2_) to prevent reoxidation of the metallic phase, which complicates system management and limits practical feasibility [30, 44, 47, 155]. Moreover, although such electrodes exhibit excellent catalytic activity toward CO_2_ adsorption and dissociation, the actual reaction typically occurs at triple phase boundaries that include a gas phase‐oxygen/proton conducting phase and an electron conducting phase. Therefore, in real applications, it is necessary to add an ion‐conducting phase to improve the reaction sites and enhance the electrochemical performance.

**FIGURE 10:**
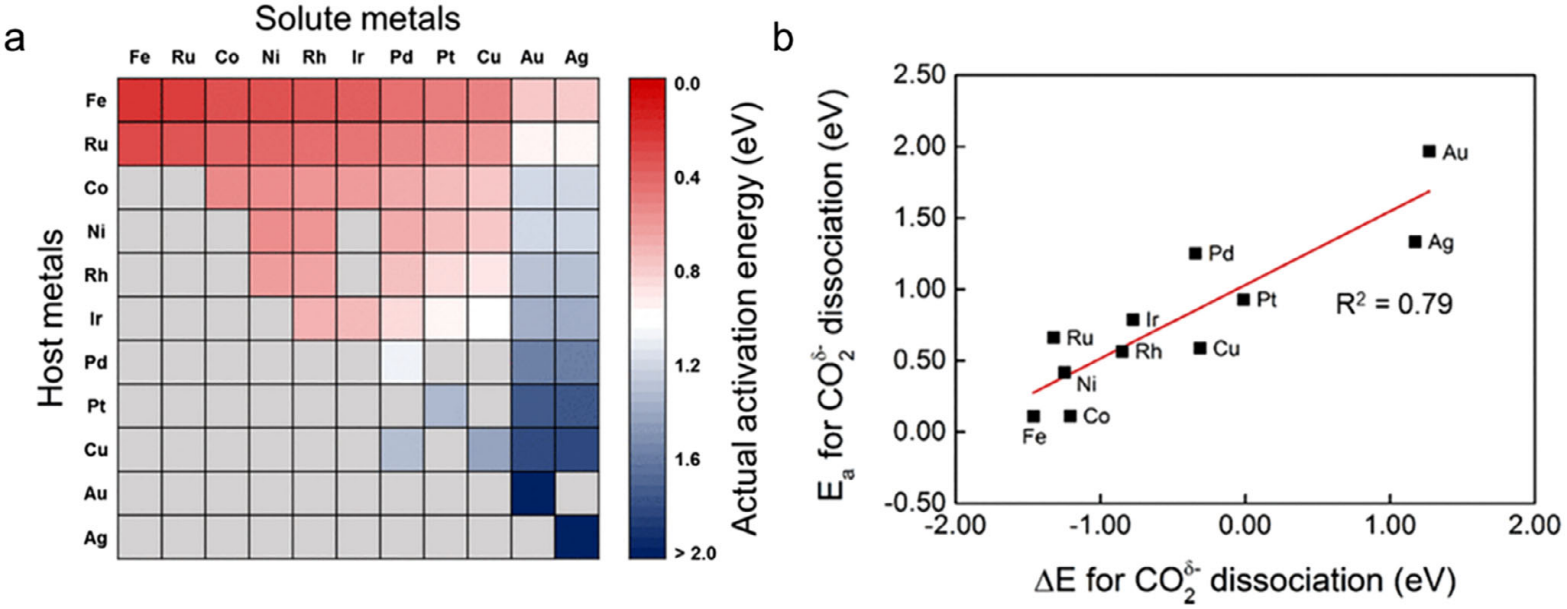
(a) Screening for Ea_act._ for CO_2_ dissociation on pure metals and bimetallic alloys. Gray cells indicate that the bimetallic alloys are not preferred for the surface segregation of solute atoms. (b) Brønsted–Evans–Polanyi relations for CO_2_^δ−^ dissociation. Reproduced with permission [157]. Copyright 2016, American Chemical Society.

The catalytic performance of metal catalysts toward CO_2_ reduction is influenced by the metal species, exposed crystal facets, and local lattice environments. First‐principles studies have demonstrated that most transition metals can thermodynamically adsorb CO_2_ through electron donation into the molecule's antibonding orbitals [156]. It further revealed that Fe possesses the highest CO_2_ dissociation capability among the investigated metals and their mixtures, with a descending order of reactivity as follows: Fe > Co > Ni > Ru > Ir > Cu > Pt > Ag > Au (Figure 10a,b) [157]. The differences in metal catalyst performance can be explained by their electronic structures and orbital occupation. Atomic electronic structures are stable when the valence orbitals are fully filled, half‐filled, or empty. For Fe, the 3d^5^ half‐filled configuration is energetically favorable; consequently, Fe tends to donate one electron to reach this stable state. This electron donation facilitates charge transfer to chemisorbed CO_2_ and activates the C═O bonds. Moreover, the binding strength between CO_2_ and metal catalysts has been found to increase with a downward shift of the metal d‐band center. A lower d‐band center promotes greater charge transfer to CO_2_, leading to stronger adsorption and enhanced activation of the molecule [158].

#### 5.2.2 Oxide Electrode

The development of oxides as cathodes significantly decreases the costs of SOERs. The major requirement for these oxides is that they should maintain stability under reducing atmospheres and should also enable concurrent conduction of oxygen ions/protons and electrons. To date, SrFeO_3‐δ‐_, LaFeO_3‐δ‐_, LaMnO_3‐δ‐_, and SrTiO_3‐δ_‐based oxides have been widely employed as SOER cathodes because of their high catalytic activity and stability.

Strategically tuning the oxygen vacancy concentration and surface basicity can significantly enhance the electrochemical performance. Surface oxygen vacancies disrupt lattice periodicity and modify the electronic structure, creating electron‐rich or electron‐deficient regions that facilitate charge transfer between the catalyst and reactants. This enables oxygen vacancies to act as active sites for CO_2_ adsorption and dissociation. Furthermore, in O‐SOERs, the dissociated oxygen species can incorporate into the electrode lattice via surface oxygen vacancies and subsequently diffuse in the bulk by hopping between adjacent oxygen vacancies within the BO_6_ octahedral framework of perovskite oxides [159]. For example, the stability of lanthanum‐based perovskites with varying doping ratios reveals a correlation between the oxygen‐vacancy formation energy and the bulk formation energy of the compound. A linear relationship was found between these two parameters in ABO_3_ perovskites (A═La and/or Be, Mg, Ca, Sr, Ba; B═Ti, V, Cr, Mn, Fe, Co, Ni). The results indicate that the oxygen‐vacancy formation energy increases linearly but inversely with the bulk formation energy [160]. The DFT calculations revealed that LaCuO_3_ exhibits the lowest oxygen vacancy formation energy, suggesting that it is the easiest to form oxygen vacancies among the LaMO_3_ series [161]. The electrochemical performance for CO_2_ electrolysis was investigated and compared among LaMO_3_ (M represents transient cations), finding that the electrochemical performance is highly dependent on the nature of the B‐site cation and follows the trend LaFeO_3_ > LaCoO_3_ > LaNiO_3_ > LaCrO_3_. DFT calculations suggested that both the nature of the B‐site cation and the presence of oxygen surface vacancies in these perovskites played a critical role in the adsorption and reduction of CO_2_ [162].

Doping the host oxide with low valence state cations and redox‐active cations favors the formation of oxygen vacancies. For instance, a Ce‐ and Co‐codoped SrFeO_3_ material, formulated as Sr_0.9_Ce_0.1_Fe_0.9_Co_0.1_O_3‐δ_, has been developed, and the corresponding cells achieved a current density of 1.30 A cm^−2^ at 1.3 V after a 48 h durability test for CO_2_ electrolysis. DFT calculations revealed that oxygen vacancies on the SrFeO_3_ surface facilitate CO_2_ adsorption [163]. Doping cations with better reducibility can promote oxygen vacancy formation. The SrTiO_3_‐based material is frequently used as a cathode for CO_2_ reduction due to its high conductivity; for example, La_0.2_Sr_0.7_TiO_3_ can reach nearly 1200 S cm^−1^ at 850°C under 10% CO/CO_2_ [164]. However, due to the stability of titanium cations, it is difficult to form oxygen vacancies, and a doping strategy is required to improve the oxygen vacancy concentration. A redox‐active Mn or Cr was introduced to the B site of the redox stable perovskite Sr_0.95_Ti_0.9_Nb_0.1_O_3_ to create oxygen vacancies after reduction for high‐temperature CO_2_ electrolysis. Compared to the bare sample in which Sr_0.95_Ti_0.9_Nb_0.1_O_3_ can be reduced to Sr_0.95_Ti_0.9_Nb_0.1_O_2.9_, a significant concentration of oxygen vacancies is additionally formed for Mn‐ or Cr‐doped samples by reducing the oxidized Sr_0.95_Ti_0.8_Nb_0.1_M_0.1_O_3_ (M═Mn, Cr) to Sr_0.95_Ti_0.8_Nb_0.1_M_0.1_O_2.85_. Consequently, the ionic conductivities of Mn‐ and Cr‐doped titanate improve by approximately 2 times compared to bare titanate in an oxidizing atmosphere and 3–6 times compared to a reducing atmosphere at intermediate temperatures [165]. Given that CO_2_ is an acidic gas, increasing the basicity of the cathode material can, in principle, improve CO_2_ adsorption. For example, A‐site doping of the alkaline cation of Cs^+^ into SrFeO_3_ accelerates the kinetics of CO_2_ adsorption and dissociation, owing to the enhanced lattice oxygen basicity arising from the low electronegativity of Cs^+^. In addition, compared with Sr^2+^, the larger ionic radius and lower valence state of Cs^+^ decrease both the formation energy of oxygen vacancies and the migration barrier of oxygen ions within the perovskite lattice. As a result, the electrolyte‐supported SOERs with the Cs_0.1_Sr_0.9_Fe_0.9_Nb_0.1_O_3‐δ_ cathode and La_0.8_Sr_0.2_Ga_0.8_Mg_0.2_O_3‐δ_ electrolyte exhibited a high current density of 2205 mA cm^−2^ at 1.6 V and 850°C and operated stably at 800°C for 100 h [166].

Beyond simple perovskites, Ruddlesden‒Popper (R‐P) oxides and double perovskites have also been widely investigated in SOERs [167]. R‐P oxides (A_n+1_B_n_O_3n+1_) possess a layered structure consisting of alternating rock‐salt layers and perovskite slabs. As n approaches infinity, the R‐P structure converges to the perovskite phase (ABO_3_). These materials typically exhibit attractive catalytic activity, high electrical conductivity, and excellent thermal and chemical stability. Currently, single (*n* = 1), double (*n* = 2), and triple (*n* = 3) layered R‐P oxides are widely investigated [167]. In O‐SOERs, single‐layer R‐P oxides such as Ln_2_NiO_4+δ_ (Ln═La, Pr, Nd) are among the most extensively studied materials [168, 169, 170]. For instance, La_2_NiO_4+δ_ ​has been employed as an oxygen electrode, demonstrating high electrochemical activity due to its favorable oxygen adsorption, dissociation, and diffusion properties [169]. More recently, strategies involving in situ exsolution have been developed to further enhance performance in R‐P oxides. For example, Fe nanoparticles exsolved from La_1.2_Sr_0.8_Mn_0.4_Fe_0.6_O_4−δ_ have been demonstrated in SOERs for CO_2_ electrolysis. The exsolved nanoparticles increase the oxygen vacancy concentration, resulting in a high current density of 1.43 A cm^−2^ at 1.5 V and 800°C [171]. Similar enhancements have been reported for Pr_1.2_Sr_0.8_Mn_0.4_Fe_0.6_O_4‐δ_, which also forms Fe nanoparticles upon reduction [172]. In addition to single‐layer R‐P oxides, multilayered R‐P compounds, such as SrEu_2_Fe_2_O_7_ ​, have also demonstrated excellent activity toward CO_2_ electrolysis. This material exhibits high catalytic activity and remarkable stability under both oxidizing and reducing atmospheres, enabling its application as both an anode and a cathode. When operated in a symmetrical SOER with a CO_2_‐CO gas mixture, a low polarization resistance of 0.48 Ω cm^2^ and a high current density of 1.27 A cm^−2^ at 1.5 V and 800°C were achieved, significantly simplifying device fabrication [173]. The double perovskite with ordered A‐site or B‐site cations was also employed as a cathode material for CO_2_ electrolysis. For example, the double perovskite Sr_2_Fe_1.5_Mo_0.5_O_6‐δ_ (SFM) with a cubic structure has been widely applied in SOER for CO_2_ electrolysis. The electrolysis performance can be further improved by compositing with the oxygen conductor Sm_0.2_Ce_0.8_O_2‐δ_. This mixed cathode promotes oxygen conduction by providing more oxygen diffusion channels, ultimately achieving a high current density [174].

In addition to cation doping, anion doping also proved to enhance the electrochemical performance toward the CO_2_ reduction reaction. A perovskite oxyfluoride electrode, Sr_2_Fe_1.5_Mo_0.5_O_6‐δ_F_0.1_ (F‐SFM), has been developed. The partial substitution of oxygen by fluorine enhances CO_2_ adsorption by approximately twofold and increases the bulk oxygen‐vacancy concentration by 35%–37% at 800°C. A single cell employing the F‐SFM cathode exhibited the highest CO_2_ electrolysis performance among reported perovskite‐based electrodes, achieving a current density of 1.36 A·cm^−2^ at 1.5 V and demonstrating excellent stability over 120 h at 800°C under harsh operating conditions [175]. Similar results have been demonstrated in Cl‐doped samples (Table 2) [176].

**TABLE 2:** Summary of CO_2_ upgrading to CO or CH_4_ using O‐SOERs and P‐SOERs.

Cathode	Electrolyte	Anode	Cathode reaction	Anode reaction	Total reaction	Current density (A cm^−2^)	Temperature (°C)	Faradaic efficiency (%)	Stability (h)	Refs.
Ni‐YSZ	YSZ	(La_0.75_Sr_0.25_)_0.95_MnO_3±δ_	CO_2_+2e^−^→CO+O^2−^	O^2−^→0.5O_2_+2e^−^	2CO_2_=2CO+O_2_	0.93@1.4 V	800	100	80	[44]
La_0.2_Sr_0.8_TiO_3+δ_‐SDC	YSZ	(La_0.8_Sr_0.2_)_0.95_MnO_3‐δ_‐SDC	CO_2_+2e‐→CO+O^2−^	2O^2−^→O_2_+4e^−^	2CO_2_=2CO+O_2_	0.125@2 V	700	36	0.5	[215]
La_0.75_Sr_0.25_Cr_0.5_Mn_0.5_O_3‐δ_‐SDC	YSZ	La_0.75_Sr_0.25_Cr_0.5_Mn_0.5_O_3‐δ_‐SDC	CO_2_+2e^−^→CO+O^2−^	2O^2−^→O_2_+4e^−^	2CO_2_=2CO+O_2_	0.18@2 V	800	55‐70	1	[216]
La_0.3_Sr_0.7_Fe_0.7_Cr_0.3_O3‐δ	YSZ	La_0.3_Sr_0.7_Fe_0.7_Cr_0.3_O_3‐δ_	CO_2_+2e‐→CO+O^2−^	2O^2−^→O_2_+4e^−^	2CO_2_=2CO+O_2_	0.41@1.6 V	800	N/A	133	[217]
Sr_2_Fe_1.5_Mo_0.5_O_6‐δ_	La_0.9_Sr_0.1_Ga_0.8_Mg_0.2_O_3‐δ_	La_0.6_Sr_0.4_Co_0.2_Fe_0.8_O_3‐δ_ ‐SDC	CO_2_+2e^−^→CO+O^2−^	2O^2−^→O_2_+4e^−^	2CO_2_=2CO+O_2_	0.71@1.5 V	800	N/A	100	[175]
Ni@Cr_2_O_3_‐YSZ	YSZ	LSM	CH_4_+2CO_2_+2e^−^ →2H_2_+3CO+ O^2−^	O^2−^→0.5O_2_+2e^−^	2CH_4_+4CO_2_═4H_2_+6CO+O_2_	0.15@2 V	800	N/A	1	[13]
Pr_0.4_Sr_1.6_(NiFe)_1.5_Mo_0.5_O_6‐δ_‐ Fe‐Ni@ FeO	LSGM	La_0.6_Sr_0.4_Co_0.2_Fe_0.8_O_3‐δ_‐Gd_0.2_Ce_0.8_O_2‐δ_	CO_2_+2e^−^→CO+O^2−^	2O^2−^→O_2_+4e^−^	2CO_2_=2CO+O_2_	1.58@1.4 V	800	40‐100	500	[14]
Sr_2_Fe_1.4_Ru_0.1_Mo_0.5_O_6‐_ *_δ_‐* RuFe@ FeOx	LSGM	BSCF‐GDC	CO_2_+2e^−^→CO+O^2−^	2O^2−^→O_2_+4e^−^	2CO_2_=2CO+O_2_	1.3@1.6 V	800	N/A	567	[16]
Sr_2_Fe_1.5_Mo_0.5_O_6‐δ_F_0.1_	LSGM	La_0.6_Sr_0.4_Co_0.2_Fe_0.8_O_3‐δ_‐SDC	CO_2_+2e^−^→CO+O^2−^	2O^2−^→O_2_+4e^−^	2CO_2_=2CO+O_2_	1.5@1.5 V	800	100	180	[176]
Sr_2_Fe_1.5_Mo_0.5_O_6‐δ_Cl_0.05_‐SDC	LSGM	La_0.6_Sr_0.4_Co_0.2_Fe_0.8_O_3‐δ_‐SDC	CO_2_+2e^−^→CO+O^2−^	2O^2−^→O_2_+4e^−^	2CO_2_=2CO+O_2_	2.05@1.5 V	800	100	500	[177]
Ni‐BZY	BZY	BZY‐ SrEu_2_Fe_1.8_Co_0.2_O_7‐δ_	CO_2_+2H^+^+2e^−^→CO (CH_4_)+ H_2_O	H_2_O→2H^+^+0.5O_2_+2e^−^	CO_2_= CO (CH_4_)+ 0.5O_2_	1.8@1.5 V	600	43	95	[30]
Ni‐BZCYYb4411	BZCYYb4411	BaCo_0.4_Fe_0.4_Zr_0.1_Y_0.1_O_3‐δ_	CO_2_+2H^+^+2e^−^→CO(CH_4_)+ H_2_O	H_2_O→2H^+^+0.5O_2_+2e^−^	CO_2_= CO (CH_4_)+ 0.5O_2_	1@1.5 V	550	40‐90	95	[205]
Pt	BZCY271	La_0.8_Sr_0.2_MnO_3‐δ_‐BZCY27	CO_2_+2H^+^+2e^−^→CO(CH_4_)+ H_2_O	H_2_O→2H^+^+0.5O_2_+2e^−^	CO_2_= CO (CH_4_)+ 0.5O_2_	0.047@2.2 V	700	39	12	[218]
Ni‐ BZCYYb1711	BZCYYb1711	BaCo_0.4_Fe_0.4_Zr_0.1_Y_0.1_O_3‐δ_	CO_2_+2H^+^+2e^−^→CO(CH_4_)+H_2_O	H_2_O→2H^+^+0.5O_2_+2e^−^	CO_2_= CO (CH_4_)+ 0.5O_2_	1.4@1.8 V	600	90	1200	[8]

*Note*: LSM: (La_0.8_Sr_0.2_)_0.95_MnO_3‐δ_; YSZ: yttria‐stabilized zirconia; LSGM: La_0.8_Sr_0.2_Ga_0.8_Mg_0.2_O_3‐δ_; BSCF: Ba_0.5_Sr_0.5_Co_0.8_Fe_0.2_O_3‐δ_; GDC: Gd_0.2_Ce_0.8_O_1.9_; SDC: Sm_0.2_Ce_0.8_O_1.9_; BZY: BaZr_0.8_Y_0.2_O_3‐δ_; BZCYYb4411: BaCe_0.4_Zr_0.4_Y_0.1_Yb_0.1_O_3‐δ_; BZCYYb1711: BaCe_0.7_Zr_0.1_Y_0.1_Yb_0.1_O_3‐δ_; BZCY271: BaCe_0.2_Zr_0.7_Y_0.1_O_3‐δ_.

#### 5.2.3 Heterogeneous Metal‐Oxide Electrode

The electrochemical reduction of CO_2_ requires catalysts with multiple functionalities for CO_2_ dissociation and oxygen ion transport. However, most simple oxides cannot simultaneously fulfill these diverse requirements. Consequently, metal‐oxide heterostructures have attracted tremendous attention for their multifunctional properties. Typically, the metallic component facilitates CO_2_ adsorption and dissociation, while defects in the host oxides, such as oxygen vacancies, provide pathways for oxygen‐ion diffusion. Two primary strategies are commonly employed to fabricate metal‐oxide electrodes: infiltration and in situ formation methods.

**FIGURE 11:**
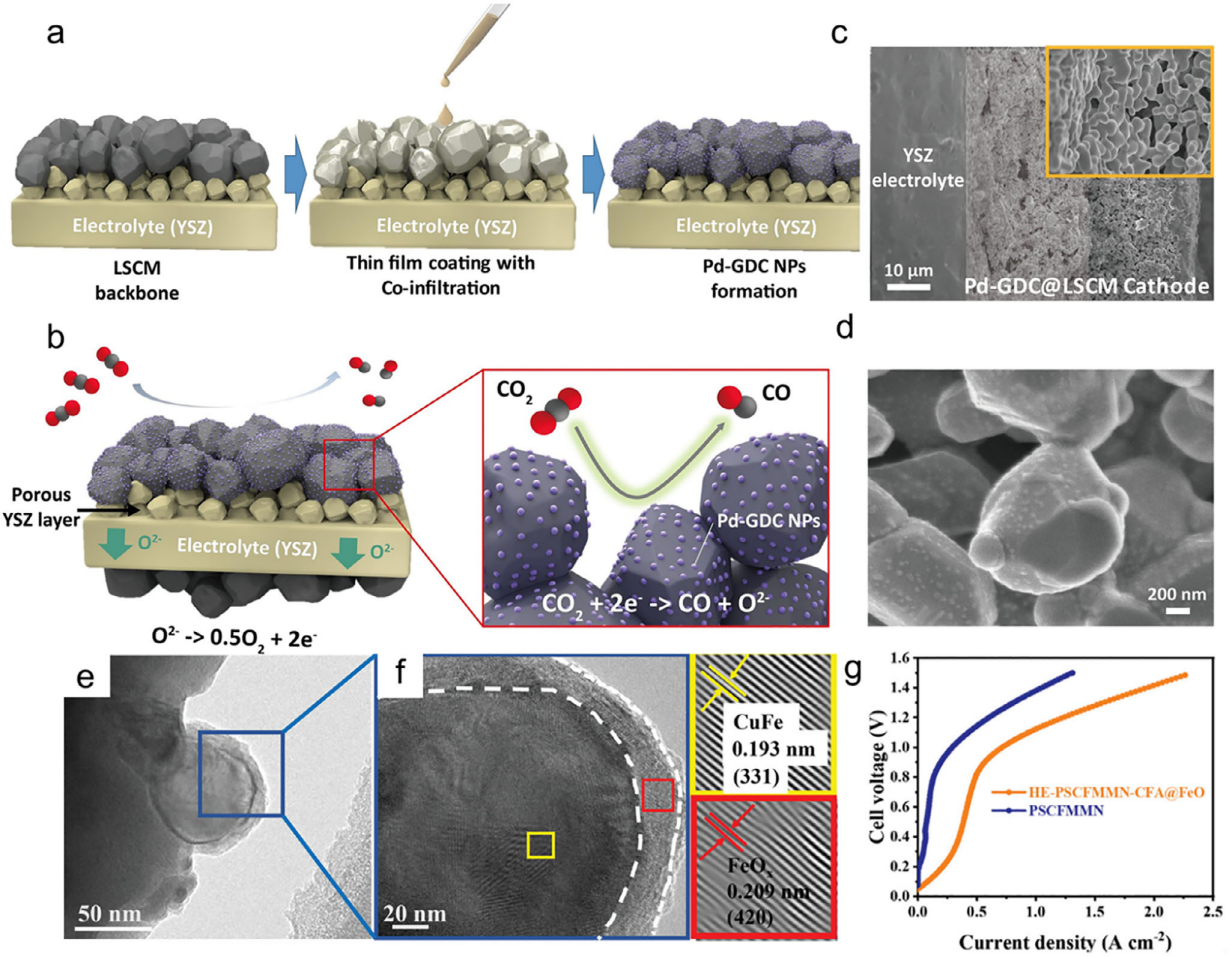
(a) Schematic of the infiltration process of Pd‐GDC@La(Sr)Cr(Mn)O_3_, (b) Schematic representation of the electrochemical CO_2_ electrolysis process in a SOER. (c) Cross‐sectional SEM images between the electrolyte and the cathode layer avoid deterioration of the cathode/electrolyte interface. (d) SEM image of the infiltrated Pd‐GDC particles. Reproduced with permission [179]. Copyright 2021, Elsevier. (e) TEM (f) images of the core–shell structured nanoparticles, high‐angle annular dark field image. (g) *I*–*V* curves of CO_2_ electrolysis performances. Reproduced with permission [94]. Copyright 2023, John Wiley and Sons.

The infiltrating method is to introduce a secondary phase that either has higher catalytic activity or enhanced stability [177, 178]. In CO_2_ electrolysis, the reactions are believed to occur at the triple phase boundaries. Therefore, increasing the number of such triple sites is expected to enhance the electrochemical performance. A previous study verified that coinfiltration of Pd and Ce_0.8_Gd_0.2_O_2_ on the La(Sr)Cr(Mn)O_3_ surface can markedly enhance the electrochemical performance due to the increased number of active triple phase boundaries and surface oxygen vacancies (Figure 11a–d) [179]. Furthermore, in preparing SOERs for commercial CO_2_ electrolysis applications, mechanical strength becomes an important consideration. Conventional metal‐oxide composite (e.g., Ni‐YSZ, Ni‐BZCYYb) electrode‐supported SOERs consisting of a porous metal‐oxide support and a dense electrolyte layer show great catalytic performance but low mechanical strength [180, 181]. Recently, it was found that replacing the metal‐oxide composite support with stainless steel can alleviate the thermal expansion mismatch between the cell and its components; however, these steels typically exhibit lower catalytic activity than Ni. To overcome this limitation, introducing a secondary catalytic phase is essential to enhance CO_2_ reduction performance. For instance, infiltrating La_0.6_Sr_0.4_Fe_0.9_Mo_0.1_O_3‐δ_ (LSFM) into the electrode has been proven to enhance performance, achieving 0.9 A·cm^−2^ at 800°C and 1.5 V. It should be noted that the infiltration amount has a significant impact on the ultimate electrochemical performance, as described by percolation theory [177]. With low loadings, LSFM particles are isolated and provide limited active sites. As the loading increases, the particles gradually form a connected network, exposing more active sites and improving catalytic performance. Nevertheless, once the optimal loading threshold is exceeded, excessive LSFM accumulation blocks gas transport pathways, leading to densely packed particle layers and ultimately deteriorating performance [182].

In addition to improving electrochemical performance, the infiltration method can also enhance operational stability. Long‐term H_2_O–CO_2_ coelectrolysis tests were conducted on both pristine Ni‐YSZ cathodes and Ni‐YSZ cathodes infiltrated with Ni‐SDC. The cell infiltrated with a 0.5 M Ni‐SDC precursor solution exhibited an initial degradation during the first 100 h, after which the performance remained relatively stable over the following 200 h. In contrast, the uninfiltrated cell suffered rapid degradation, exhibiting a 73% performance loss within 50 h. This improvement is attributed to the ability of Ni‐SDC infiltration to suppress Ni oxidation and Ni particle loss during coelectrolysis, thereby preserving the continuous Ni electronic conduction network within the electrode [47].

To simplify the nanoparticle formation process and prevent nanoparticle coarsening, the in situ formation of nanoparticles from oxide precursors has been widely investigated. The in situ exsolution process can be triggered by intrinsic material properties (e.g., dopant properties, lattice strain, electrostatics) or external annealing conditions (e.g., temperature, oxygen partial pressure, water partial pressure, electrical bias) [82, 130]. Typically, reducible elements such as Ni, Fe, and Co are first doped into host oxides, and the material is then exposed to reducing atmospheres to induce lattice oxygen exsolution and lattice strain, thereby triggering the exsolution of nanoparticles, which are typically on the order of hundreds of nanometers in size. The in situ exsolution method has the advantages of high catalytic activity and simple processing. Owing to the anchoring effect of nanoparticles to host oxides, nanoparticles usually exhibit better resistance to coarsening, thus enabling improved stability for electrocatalytic performance under hydrocarbon fuels [183]. Furthermore, with the coexsolution of lattice oxygen to the surface, the host oxide surface typically exhibits a higher concentration of active oxygen species and modified electronic structures, thereby enhancing catalytic performance [184]. This strategy has been used not only in SOER but also in fuel cells and thermal catalysts [82, 138, 183, 185]. The exsolution process can be affected by cation deficiencies; in particular, A‐site deficiency promotes B‐site cation exsolution upon reduction. For example, doping the B‐site with transient cations while creating an A‐site deficiency and formulating it as La_0.4_Sr_0.4_M_0.06_Ti_0.94_O_3‐δ_ (M = Fe^3+^ or Ni^2+^) triggers nanoparticle exsolution in the SOER. The exsolution is attributed to the limited ability of the host lattice to accommodate defects, resulting in the formation of abundant Fe^0^, Ni^0^, and oxygen vacancies that dramatically enhance the electrolysis performance [185]. Another example is developing a series of Sr_x_Ti_0.7_Cu_0.2_Mo_0.1_O_3‐δ_ (SxTCM, x = 1, 0.975, 0.95) perovskite oxides as cathodes for SOER. By tuning the defects in the perovskite A‐site, abundant oxygen vacancies are formed in the perovskite lattice, which effectively improves the oxygen ion transport ability and acts as the active sites for the CO_2_ molecule. Furthermore, uniformly dispersed Cu nanoparticles on the STCM substrate are generated in situ, leading to the formation of abundant active sites for the CO_2_ reduction reaction [186].

In addition to intrinsic material properties, the in situ exsolution process can also be influenced by external reaction conditions. The as‐prepared oxide materials stabilize in a thermodynamically favored phase. However, changes in the external environment perturb the defect chemistry and phase stability of the host lattice, thereby driving the segregation and nucleation of secondary phases via exsolution. For example, a small amount of nickel can be well dissolved into SrFe_1.5_Mo_0.5_O_6_ and form a cubic perovskite structure in air, yet the reduction of this material at 800°C under H_2_ triggers the formation of Ni‐Fe alloys over the material surface [138]. Similarly, the prereduction of Pr_0.8_Sr_1.2_(CuFe)_0.4_Mo_0.2_Mn_0.2_Nb_0.2_O_4‐δ_ triggers the formation of core‐shell structured CuFe alloy@FeO_x_ (Figure 11e,f), and in situ XPS and DFT calculations confirm that the exsolution of these core‐shell nanoparticles introduces additional oxygen vacancies within the host oxide substrate, acting as active reaction sites for CO_2_ electrolysis and significantly enhancing the electrochemical performance (Figure 11g) [94]. During SOER operation, the cathode undergoes reduction reactions. Applying a high voltage for a very short duration can induce deep cathodic reduction, thereby promoting nanoparticle exsolution in the cathode material. For example, applying an electrical bias over a fuel cell with a La_0.43_Ca_0.37_Ni_0.06_Ti_0.94_O_3‐δ_ electrode for 2 V for a few seconds yields small but high‐density nickel nanoparticles, which dramatically enhance the electrochemical bias [137]. Similarly, applying voltage over Sr_2_Fe_1.5+x_Mo_0.5_O_6‐δ_ for 180 s constructed a metal‐oxide interface with high activity and stability, which not only prevents carbon deposition but also prevents sintering during CO_2_ electrolysis [187].

However, it should be noted that strategically formed nanoparticles, whether generated via infiltration or in situ exsolution, may still undergo coarsening during long‐term operation. At elevated temperatures, infiltrated nanoparticles coarsen as a result of surface energy minimization and are driven by particle migration, coalescence, and atomic diffusion processes [188]. For in situ exsolved nanoparticles, although the host oxide can partially restrain particle growth [10, 189], prolonged exposure to high temperatures can still lead to particle enlargement, resulting in decreased electrocatalytic performance [190]. Previous studies have employed modeling approaches to investigate the exsolution and growth behavior of nickel from La_0.4_Sr_0.4_Sc_0.9_Ni_0.1_O_3‐δ_. The results suggest that size‐dependent strain and the limited availability of Ni are key factors governing the particle growth rate [190]. Nanoparticle aggregation reduces the specific surface area and porosity, ultimately deteriorating the electrochemical performance. Such aggregation can be effectively mitigated by operating under lower temperatures and milder reaction conditions, as well as through rational material design and the selection of appropriate infiltration precursors [177, 191, 192].

#### 5.2.4 Tailoring Electrode Microstructure

**FIGURE 12:**
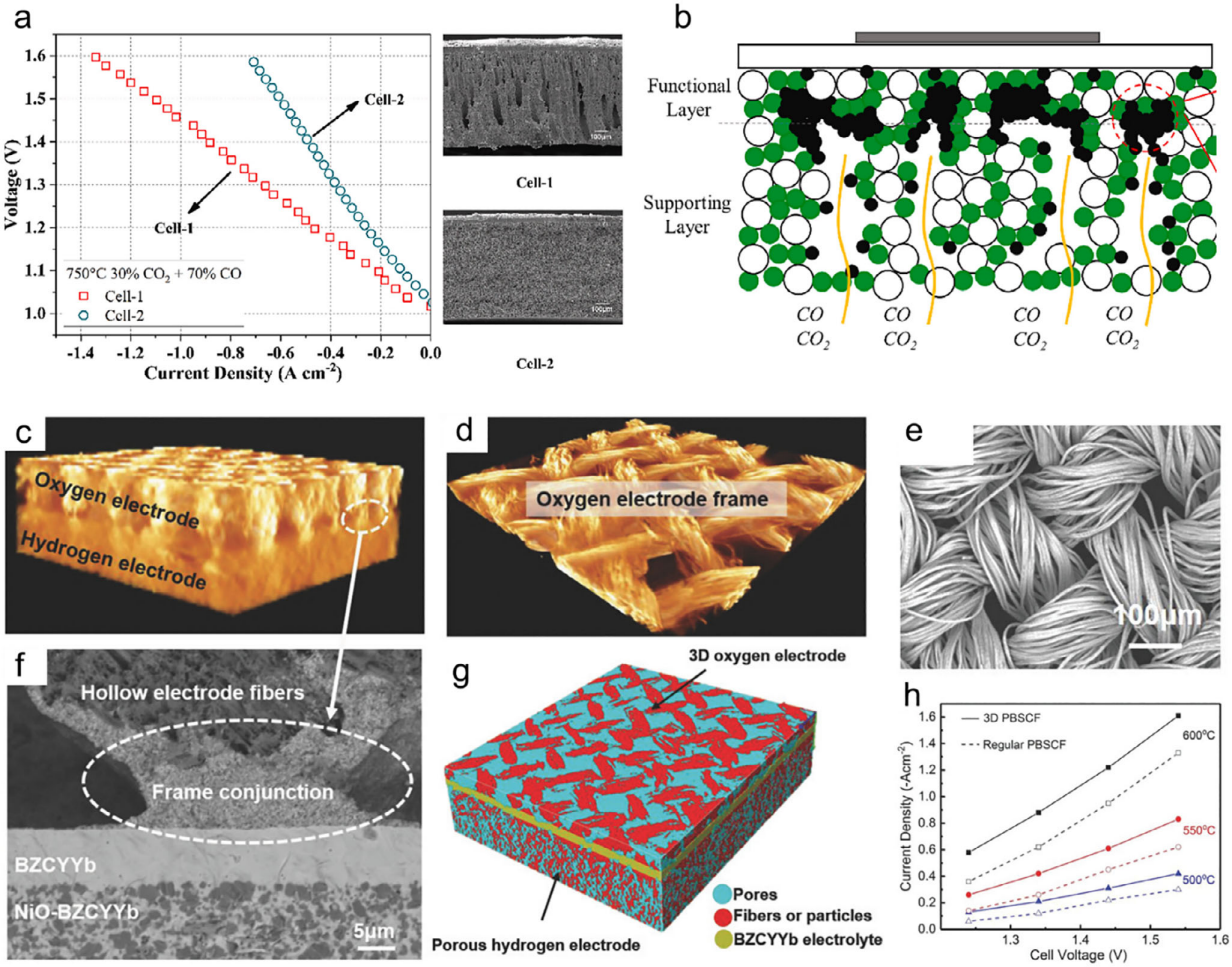
(a) Comparison of SOER performance with straight pores (Cell 1) and tortuous pores (Cell 2) in the cathode. The corresponding SEM images are shown on the right. (b) Schematic illustration of carbon deposition within straight pores in the SOER cathode. Reproduced with permission from [195], copyright 2022, Elsevier. (c) 3D X‐ray microscopy image of the cell consisting of a hydrogen electrode (bottom layer), electrolyte (invisible), and 3D steam electrode (top layer). (d) The bulk electrode frame with hierarchical gaps, which would facilitate fast mass transfer. (e) SEM image of the cross section of the cell showing the contact area between the steam electrode frame and electrolyte, and (f) SEM image of a sintered 3D PBSCF framework surface. (g) Reconstructed 3D image for the cell with dimensions of 0.4 × 0.4 × 0.1 mm^3^. (h) Electrolysis performance enhanced by the SAUP 3D PBSCF steam electrode compared with that of the conventional PBSCF electrode at different temperatures. Reproduced with permission [200], under the terms of the Creative Commons CC BY license.

Electrode performance can be enhanced through microstructural tailoring, which includes optimization of pore size and tortuosity to promote gas diffusion while accommodating a high density of nanocatalysts and the design of advanced architectures to improve mass transport. Ni‐based composites are widely used in SOERs for CO_2_ electrolysis, primarily due to their low cost, ease of cell‐shape modulation, and high electronic conductivity. For example, Ni‐YSZ composites can achieve a total conductivity of approximately 1000 S·cm^−1^ when the nickel volume fraction exceeds 30% [193]. However, conventionally pressed cathodes often suffer from slow gas diffusion and limited reactive sites, mainly caused by high‐temperature sintering that reduces porosity and active surface area. To address these issues, the fabrication of electrodes with controlled pore structures was studied. For example, a high‐performance SOER was developed by integrating SrSc_0.175_Nb_0.025_Co_0.8_O_3‐δ_ (SSNC) with a phase‐inversion tape‐cast Ni‐YSZ electrode. The Ni‐YSZ electrode fabricated using this method dramatically improved the porosity to 60.8% [194]. During CO_2_ electrolysis, cathodes with straight pores exhibit enhanced electrochemical performance by facilitating gas diffusion (Figure 12a). Moreover, such pore architectures have been demonstrated to suppress carbon deposition by enabling the rapid removal of CO from reaction sites (Figure 12b), and straight pores provide sufficient free volume to accommodate the growth of small carbon without inducing mechanical damage or rupture of the cell [195]. To avoid pore structure collapse and achieve controllable pore structures, the combination of freeze‐drying tape casting with impregnation methods was investigated. These electrodes feature straight channel‐like pores, with nano‐ or submicron‐sized catalyst particles homogeneously coated onto the surfaces of the porous scaffold. The low tortuosity factor (∼1.3) and high porosity (∼50 vol%) significantly facilitate diffusion, while the finely dispersed catalysts enhance electrode reaction kinetics. As a result, the cell delivers a high current density of 0.8 A·cm^−2^ at 1.30 V when coelectrolyzing H_2_O and CO_2_ at 800°C [196]. In addition to Ni‐based cathodes, redox‐stable perovskite oxides, such as La_0.8_Sr_0.2_Cr_0.5_Fe_0.5_O_3‐δ_, have also been fabricated via phase‐inversion tape casting. However, direct application of this oxide yields limited CO_2_ electrolysis performance due to insufficient catalytic performance. To overcome this drawback, Sr_2_Fe_1.5_Mo_0.5_O_6‐δ_ was infiltrated into the straight pore channels, which markedly enhanced the surface reaction kinetics. As a result, the SOEC delivered a current density of 2 Acm^−2^ at 2.0 V and 800°C [197].

It should be noted that the introduction of large, straight pores can, in some cases, reduce the active reaction zone near the electrode/electrolyte interface, which is critical for many electrochemical reactions. To address this issue, the strategic fabrication of a relatively dense functional layer using tape‐casting techniques has proven effective. For example, multilayer tape casting with a low content of pore formers can be employed to construct a functional layer adjacent to the electrolyte, thereby significantly enhancing electrochemical performance [198]. Another approach involves combining phase inversion with tape casting. By exploiting the difference in phase inversion rates across the two sides of the film, a relatively dense layer can be formed near the electrolyte, which has been shown to effectively improve electrochemical performance under hydrocarbon fuel conditions [199].

In addition to developing electrodes with high porosity, addressing the mass transfer also proved effective in enhancing the electrochemical performance. To address this, a self‐architectured ultra‐porous (SAUP) 3D steam electrode was developed for efficient P‐SOER for operating below 600°C (Figure 12c–g). The electrode was fabricated using the fabric textile coupon template method, followed by catalyst infiltration, calcination, and bonding to the electrolyte. This electrode significantly promoted mass transfer and gas diffusion, resulting in an enhanced electrolysis current density from 500°C to 600°C (Figure 12 h). Interestingly, performance enhancement is observed during electrolysis at an applied voltage of 1.6 V at 500°C for over 75 h, which is attributed to the “bridging” effect from reorganization of the steam electrode [200].

### 5.3 Move Beyond CO_2_ Electrolysis, Upgrade CO_2_ and H_2_O Into Value‐Added Hydrocarbons

**FIGURE 13:**
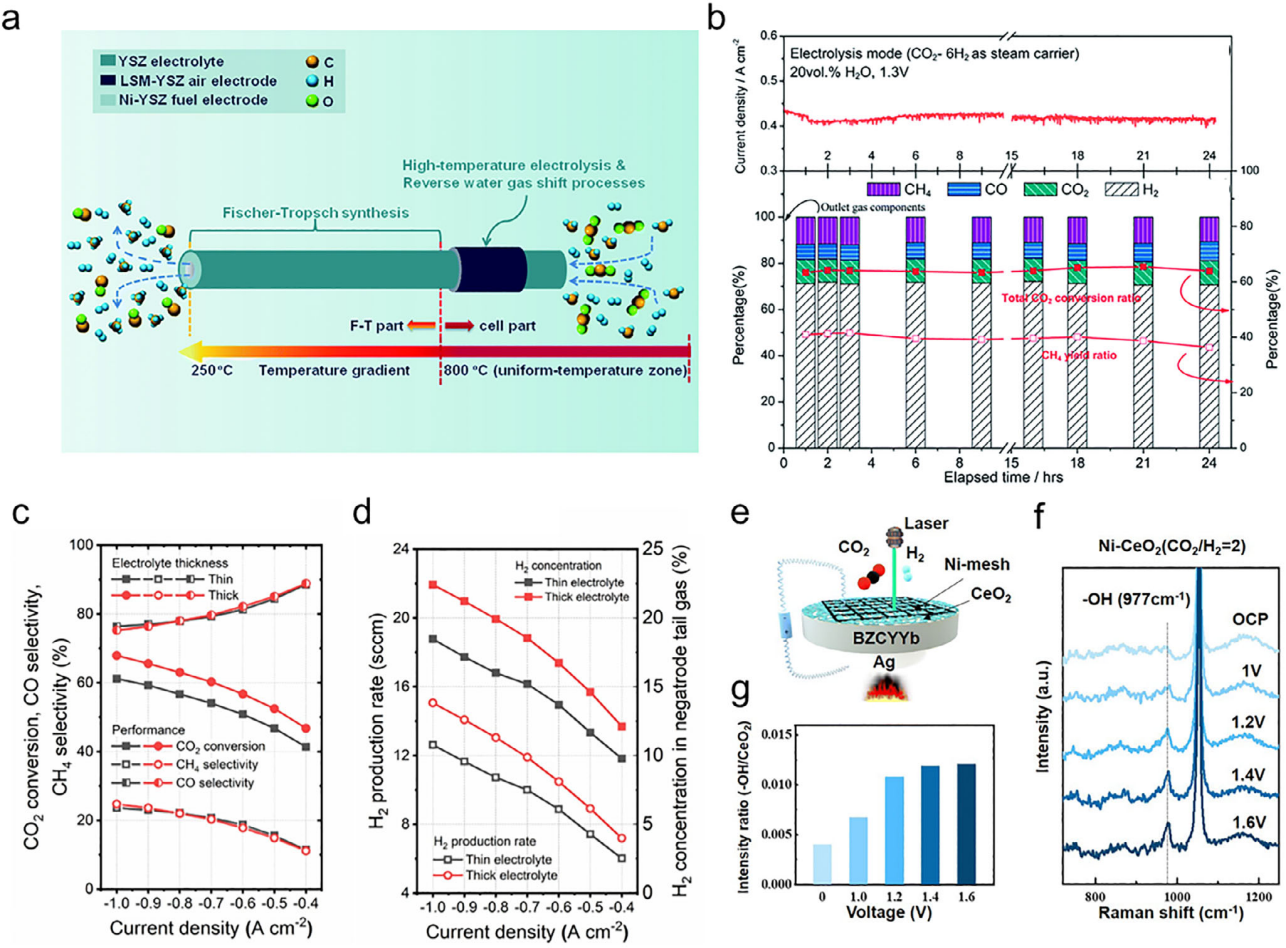
(a) Illustration of direct methane synthesis from CO_2_–H_2_O coelectrolysis in a tubular SOER combining high‐temperature electrolysis and a reduced temperature Fischer‐Tropsch reactor. (b) Performance of a tubular unit operated at 1.3 V. The inlet steam carrier is ∼15 mL min^−1^ CO_2_–H_2_ with a relative humidity of 20%. Reproduced with permission [115]. Copyright 2014, Royal Society of Chemistry. (c) H_2_‐to‐CO_2_ ratios on CO_2_ conversion and CH_4_ selectivity in H_2_O‐CO_2_ coelectrolysis; (d) Effect of electrolyte thickness on the hydrogen production rate and the hydrogen concentration in the exhaust gas of the negatrode. Reproduced with permission [204]. Copyright 2025, Elsevier. (e) Schematic of the in situ Raman spectra measurement on SOERs with the CeO_2_‐modified electrode, in which the Ni mesh is embedded into the electrode. (f) In situ Raman spectra obtained near the Ni–CeO_2_ interface in a mixture of CO_2_ and H_2_ (5% CO_2_ + 2.5% H_2_) at 550°C under an applied potential ranging from 1 to 1.6 V. g, Intensity ratio of the surface hydroxyl (‐OH) to CeO_2_ F_2g_ peak in the in situ Raman spectra in (f). Reproduced with permission [205]. Copyright 2023, Royal Society of Chemistry.

Coelectrolysis of CO_2_ and H_2_O can generate CO‐H_2_ syngas, which can be used as feedstock for hydrocarbon synthesis via F‐T synthesis of nCO + (2n+1)H_2_→C_n_H_2n+2_+nH_2_O [201, 202]. Methane formation is a feasible reaction in nickel‐based electrodes and can be realized in both O‐SOER and P‐SOERs. Thermodynamically, methane formation from CO‐H_2_ syngas is favored at lower temperatures, whereas higher temperatures stabilize CO, making such a reaction more suitable in P‐SOERs. For example, in an O‐SOER employing Ni‐YSZ|ScSZ|LSM‐ScSZ, only when a very high electrolysis voltage at 2 V can the formation of methane be triggered, and the C+2H_2_→CH_4_ reaction is proposed as the reaction pathway for methane production [203]. To achieve high CH_4_ yields while maintaining high electrochemical performance, Chen et al. fabricated tubular‐shaped O‐SOERs and utilized different temperature zones in a furnace. The higher temperature zone of 800°C favors the electrochemical production of CO‐H_2_ syngas, and when this syngas passes to the lower temperature zone (250°C), it will thermally catalytically form CH_4_ (Figure 13a). This method enables a methane concentration of approximately 12% over 24 h of continuous CO_2_‐H_2_O coelectrolysis (Figure 13b) [115]. Methane formation is more readily achieved in P‐SOERs, and CH_4_ formation is affected by combined factors, including temperature, current density, and electrolyte thickness. It has been reported that lower operating temperatures and higher current densities improve CH_4_ selectivity [8, 30, 204]. In addition, the electrolyte thickness also plays a critical role in determining the efficiency. Thicker electrolytes effectively depressed undesirable electron conduction and facilitated greater proton accumulation at the cathode, favoring H_2_ production and promoting CO_2_ reduction (Figure 13c,d) [204].

To enhance CH_4_ production in P‐SOERs, rational electrode surface design is an effective strategy. One study demonstrated that impregnating a thin CeO_2_ layer onto the BaZr_0.1_Ce_0.7_Y_0.1_Yb_0.1_O_3‐δ_ surface of a Ni‐ BaZr_0.1_Ce_0.7_Y_0.1_Yb_0.1_O_3‐δ_ cathode can promote CH_4_ formation. The resulting CeO_2_‐modified electrode delivered more than three times higher CH_4_ selectivity at 550°C than the unmodified electrode. In situ Raman spectroscopy further reveals how the applied external voltage regulates proton availability and promotes CH_4_ formation. At lower voltages, the kinetics of ─OH formation and CO_2_ hydrogenation are limited by the number of protons pumped across the electrolyte, resulting in a strong voltage dependence for ─OH species. In contrast, at higher voltages, sufficient protons are already supplied from the counter electrode, and the reaction becomes constrained by the availability and intrinsic activity of surface reactive sites, showing less dependence on voltage (Figure 13e,f) [205].

Beyond methane synthesis, syngas can also be converted into higher hydrocarbons through surface polymerization of CH_x_ (x = 1, 2, or 3) intermediates. Numerical studies have shown that the distribution of C_1_–C_5_ products in F‐T reactors can be regulated by adjusting the H_2_O/CO_2_ ratio during the electrolysis process [206]. Nevertheless, the resulting products typically follow a statistical distribution described by the Anderson‐Schulz‐Flory (ASF) model, which inherently limits the selectivity toward C_2_–C_4_ hydrocarbons (including both alkenes and paraffins) to approximately 58% while yielding an undesired methane fraction of approximately 29% [207]. This limitation can be effectively mitigated by precisely controlling C–C coupling reactions while suppressing overhydrogenation and methane formation, an approach that can be realized using bifunctional catalysts featuring two complementary types of active sites. For example, in ZnCrO_x_‐based zeolite catalysts, the partially reduced oxide surface activates CO and H_2_, whereas subsequent C–C coupling occurs within the confined acidic pores of the zeolite framework, promoting the formation of higher hydrocarbons [201].

Realizing the commercialization of SOERs for value‐added chemical production requires improved operational stability and reduced operating temperatures. Techno‐economic analyses have evaluated the coelectrolysis of H_2_O and CO_2_ integrated with F‐T synthesis to produce diesel [208], gasoline [209], liquid fuels [210], waxes [211], methanol [212], and other electrofuels (Table 3). These studies show that stack cost is the dominant contributor to overall investment and imposes stringent requirements on stack lifetime (>10,000 h) [213]. From a technological perspective, the major challenges hindering the integration of F‐T synthesis into SOERs arise from the mismatch in operating temperatures, low overall efficiency, and the lack of efficient and reliable catalysts. Low‐temperature F‐T synthesis for producing light olefins and waxes typically operates at 200–250°C using cobalt‐based catalysts, whereas high‐temperature F‐T synthesis for generating light alkanes, olefins, and gasoline occurs at 300–350°C with iron‐based catalysts. However, both regimes remain significantly below the typical operating temperature of SOERs, which generally exceed 450°C. These findings highlight the necessity of developing advanced electrode and electrolyte materials capable of sustaining stable SOER operation below 350°C and indicate the application potential of P‐SOERs for in situ integration with F‐T synthesis.

**TABLE 3:** Summary of techno‐economic evaluations for high‐hydrocarbon production by integrating SOER with F‐T synthesis.

Production	Stack cost	Stack lifetime (h)	Techno‐economic costs ($/l)	Year	Refs.
Diesel	491€/kW	8000/year	1.85€/l	2022	[218]
Electrofuel	1725$/kW	>144 000	0.83–5.3$/l	2021	[219]
Gasoline	3300‐3850$/kW	48 000	2.2–2.54$/l	2019	[220]
Waxes	769$/kW	72 000	2.34$/l	2018	[211]
Liquid fuel	/	/	1.08–3.67$/l	2016	[210]
Gasoline and diesel	200$/kW	/	15.42–16.66$/l	2012	[209]
Diesel	438$/kW	>30 000	1.26–2.42$/l	2010	[208]

## 6 SOER for Anodic Hydrocarbon Upgrading

### 6.1 Reaction Mechanisms

Light olefins (e.g., ethylene and propylene) are essential feedstocks for the production of polymers, oxygenates, and many chemical intermediates. Conventionally, steam cracking and fluid catalytic cracking of naphtha, light diesel, and other oil‐derived byproducts have been the dominant industrial routes to produce these olefins [86]. However, these processes require extremely high temperatures and substantial energy input, with significant carbon emissions [72, 86]. With the shale gas revolution, the cost of natural gas production has decreased substantially in some regions (e.g., in the United States, natural gas prices dropped by approximately 50–75% compared to 2005). Although global natural gas prices have fluctuated in recent years, the overall economics still make the direct conversion of natural gas to syngas or value‐added olefins attractive pathways. However, methane is a highly stable molecule, requiring a high energy of 439 kJ·mol^−1^ to break the first C‐H bond. In addition, it lacks a dipole moment and low polarizability, requiring a strong external electric field to polarize and activate methane [221].

The oxidative coupling of methane (OCM) and nonoxidative coupling of methane (NOCM) are critical technologies for upgrading methane into olefins and aromatics. From a thermodynamic perspective, OCM is more favorable, whereas NOCM requires substantially higher energy at temperatures above 950°C. Achieving the desired products critically depends on precise control of the oxygen supply or proton extraction during the reaction, as well as the development of efficient catalysts capable of selectively producing target products. For example, OCM often suffers from overoxidation, leading to the formation of CO and CO_2_, which significantly reduces olefin yields. In contrast, NOCM tends to favor the formation of graphitic carbon rather than olefins, resulting in limited olefin selectivity and rapid catalyst deactivation [222, 223]. In addition to methane, other light alkanes, such as ethane (C_2_H_6_) and propane (C_3_H_8_), have also been used for producing light olefins. These hydrocarbons have lower dehydrogenation energies than methane (e.g., 423 kJ·mol^−1^ for C_2_H_6_→C_2_H_5_•+H• and 401 kJ·mol^−1^ in C_3_H_8_ →iC_3_H_7_•+H• or 423 kJ·mol^−1^ in C_3_H_8_ →n‐C_3_H_7_•+H•), requiring fewer energy inputs.

**FIGURE 14:**
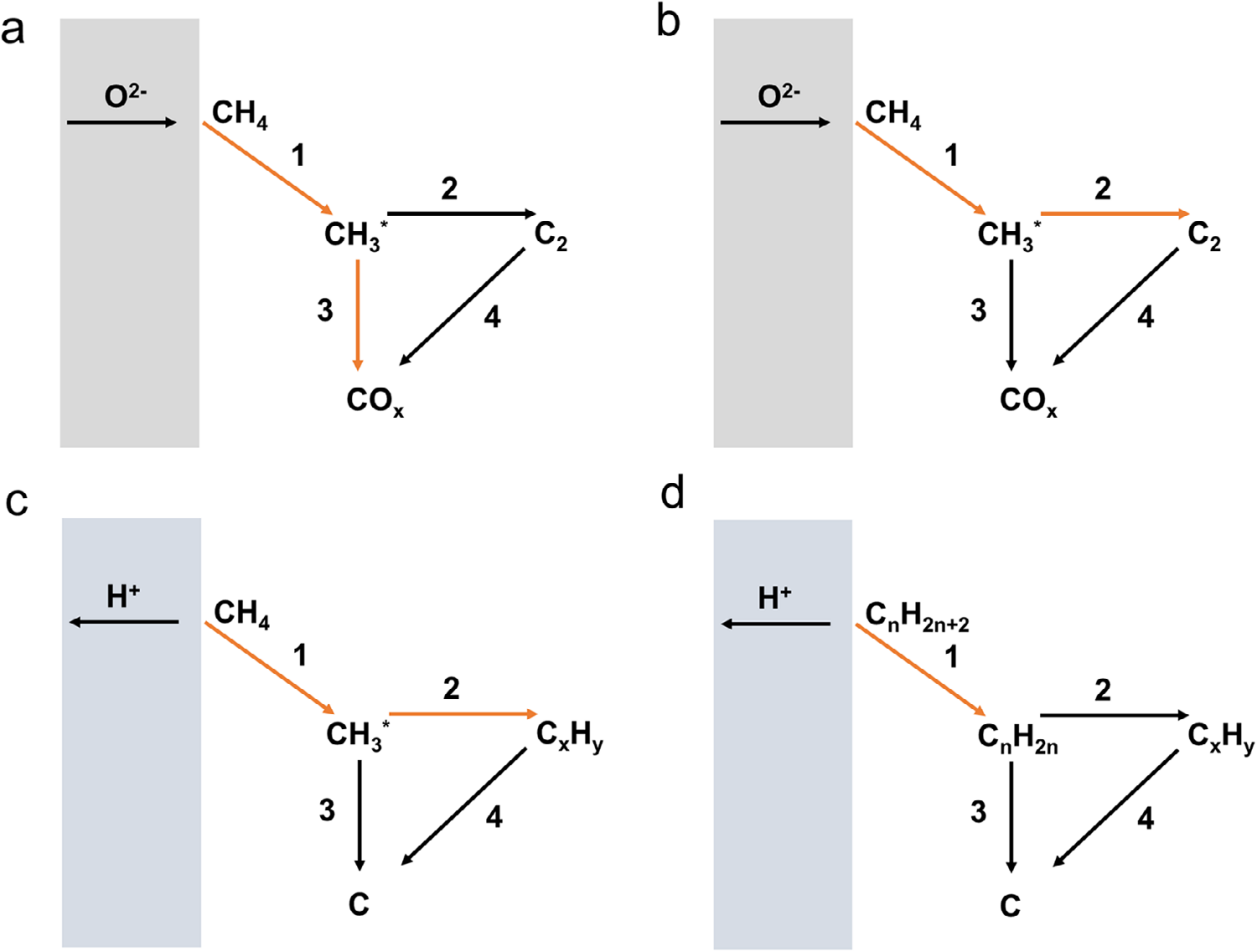
Schematic illustration of (a) methane oxidation to syngas in O‐SOER. (b) Methane oxidative coupling to C_2_ products in the O‐SOER. (c) Nonoxidative coupling of methane in P‐SOER. (d) Nonoxidative dehydrogenation of alkanes to olefins in the P‐SOER. The orange arrow indicates the preferred reaction pathway.

The unique advantage of SOERs for OCM lies in their precisely controllable reaction kinetics. Both the oxygen supply and hydrogen extraction can be finely regulated by adjusting the applied electrical bias, thereby enhancing product selectivity while simultaneously suppressing carbon deposition. In the case of methane oxidation to syngas (Figure 14a), the reaction must be carefully controlled to selectively produce CO and H_2_ while preventing methyl coupling or overoxidation to CO_2_. The resulting H_2_‐CO syngas can be directly utilized for chemical synthesis or as a feed gas for solid oxide fuel cells. For olefin production via OCM in SOERs, achieving high selectivity requires both precise control over the oxygen species flux and the development of highly selective catalysts (Figure 14b) for carbon‒carbon coupling. However, the oxidative process releases heat, which reduces the calorific value of the chemical products. Moreover, if the oxidation process is not properly controlled, the formation of thermodynamically stable species such as H_2_O and CO_2_ can lead to significant elemental loss. Therefore, it is crucial to efficiently utilize this self‐generated heat to compensate for the cathodic reactions, thereby achieving high overall energy efficiency.

In contrast, during NOCM to aromatics and alkane dehydrogenation to olefins, SOER operation tends to promote the formation of solid carbon or hydrocarbons with undesirably high C/H ratios, leading to catalyst deactivation and limited selectivity (Figure 14c,d). Furthermore, oxidative alkane dehydrogenation is an exothermic reaction; thus, the heat demand of the SOER for the electrolysis of CO_2_ or H_2_O at the cathode can be compensated by the anodic waste heat of the OCM process, potentially achieving thermally neutral conditions.

### 6.2 Methane to Syngas

**FIGURE 15:**
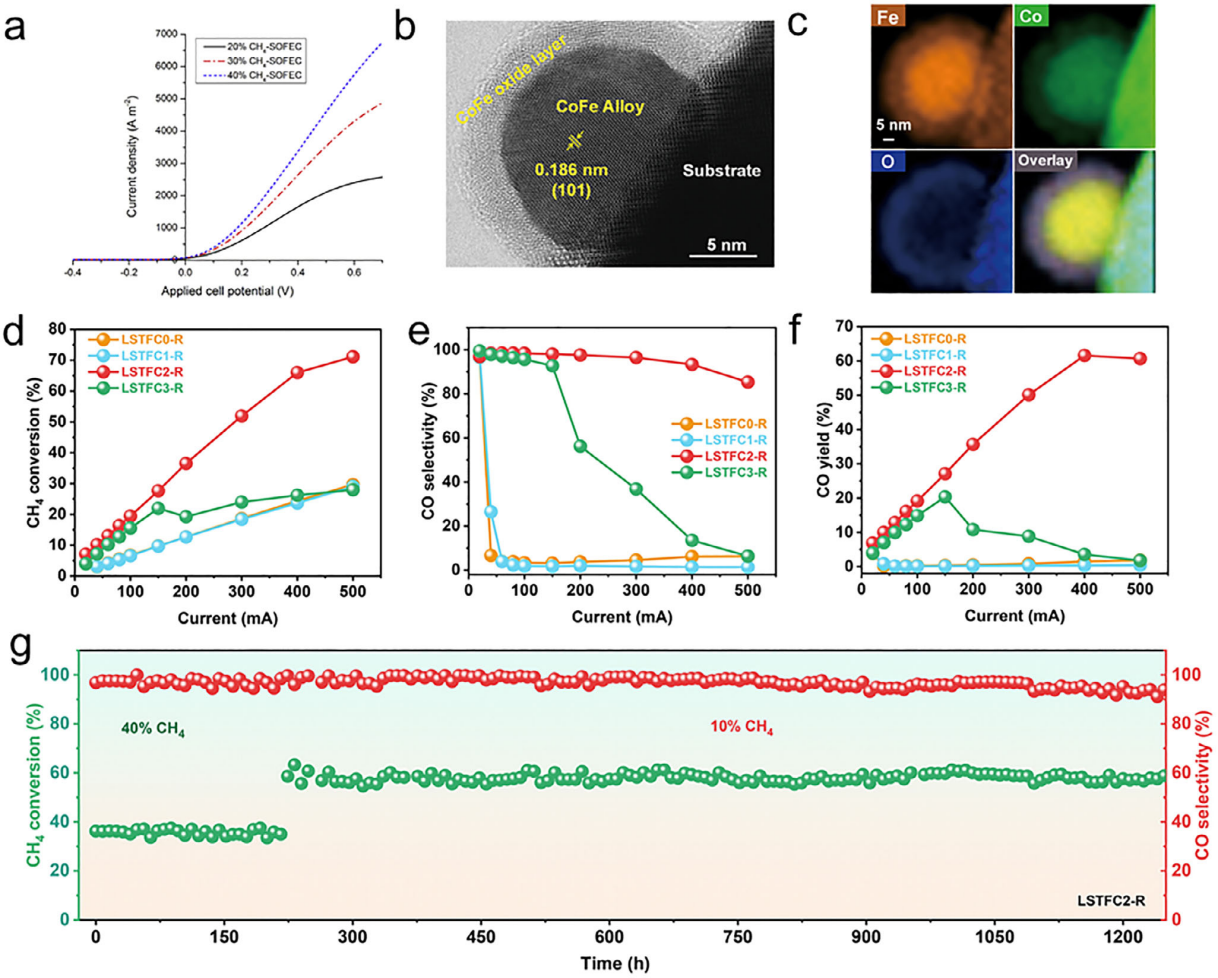
(a) Effects of inlet methane mole fraction on CH_4_‐SOER performance at 1073 K. Reproduced with permission from [22], copyright 2016, Elsevier. (b) TEM and (c) EDS mapping results of La_0.6_Sr_0.4_Ti_0.3_Fe_0.5_Co_0.2_O_3‐δ_ with Fe‐Co alloy exsolution. Methane oxidation performance of (d) CH_4_ conversion, (e) CO selectivity, and (f) CO yield for LSTFCx‐R (x = 0–3) with different currents at 800°C. (g) stability test of LSTFC for the anodic methane oxidation reaction. Reproduced with permission [27]. Copyright 2024, Elsevier.

The syngas composing CO and H_2_ is an intermediate product in the coal and petrochemical industries and has been widely applied in many fields, including ammonia [224], methane [225], methanol [226], dimethyl ether [227], and olefin [207] production. Methane can be oxidized to syngas using O‐SOER at the anode (Equation 7), while the cathode usually undergoes a CO_2_ reduction reaction and forms CO (Equation 8) or water electrolysis to form H_2_ (Equation 9). When coupling water electrolysis with methane oxidation to syngas, modeling calculations found that the overall energy demand is significantly reduced compared with conventional operation in which the anode is fed with air. This improvement is attributed to the exothermic nature of methane oxidation, which elevates the local temperature and/or thermodynamically promotes anodic oxidation reactions. In experimental validations, reaching the same current density, the anode fed with methane can decrease the voltage by approximately 0.8 V at 850°C [228]. Furthermore, a two‐dimensional model was developed to examine the influence of methane‐assisted operation on CO_2_/H_2_O coelectrolysis, finding that methane participation effectively lowers the equilibrium potential of the O‐SOER and significantly reduces the electrical energy required for coelectrolysis. When operating in fuel cell mode, the application of CH_4_ as fuel in O‐SOER is capable of simultaneously generating electrical power and producing syngas at current densities below 600 A m^−2^ and at 1123 K, and the performance increases with increasing methane content (Figure 15a). Moreover, unlike conventional SOERs, whose performance shows minimal dependence on the anode gas flow rate, the methane‐assisted SOER exhibits strong sensitivity to anodic gas flow [22]. In addition, the operation pressure also influences the electrochemical performance and product yields. For example, a pressurized R‐SOC reactor was designed to test microtubular R‐SOCs at 650°C on a Ni‐YSZ/ScSZ/LSM‐ScSZ tubular cell. It was found that the CH_4_ mole ratio reaches a maximum of 10% at OCV and 25% at 1.3 V at 10 atm. The competition between the methanation reaction and electrolysis is found to control the overall methane production [229].

(7) $$CH_{4}+O^{2}\to \mathrm{CO}+2H_{2}+2e^{-}$$

(8) $$CO_{2}+2e^{-}\to \mathrm{CO}+O^{2-}$$

(9) $$H_{2}O+2e^{-}\to H_{2}+O^{2-}$$

Although studies have proven that incorporating methane into the reaction chamber can shift the reaction to anodic methane oxidation, problems remain concerning the sluggish anode reaction due to the stability of methane molecules, thus requiring high‐performance catalysts to activate methane. Similar to cathode catalysts, developing heterogeneous bifunctional catalysts is highly beneficial; for example, a double perovskite oxide Sr_2_Fe_1.4_Pt_0.1_Mo_0.5_O_6‐δ_ was synthesized as both an electronic conductor and a catalyst for methane oxidation at the O‐SOER anode. The high‐temperature treatment under a reducing atmosphere results in platinum nanoparticles exsolving from the perovskite lattice and uniformly dispersing on the oxide surface. These exsolved Pt nanoparticles significantly improve the catalyst's ability to adsorb and dissociate methane molecules. This resulted in a high electrochemical performance of 0.85A cm^−2^ at 1.4 V when the anode was fed with methane, while the cathode was fed with H_2_O at 850°C, which is a 102.4% enhancement compared to the undoped SFM cell [230]. In terms of pairing CO_2_ electrolysis and methane oxidation in O‐SOERs, the methane oxidation activity was investigated by tailoring the exsolution energy of B‐site components through optimizing the Co/Fe ratio to control the particle size and exsolved nanoparticle density (Figure 15b,c). A series of catalyst formulations, such as La_0.6_Sr_0.4_Ti_0.3_Fe_0.7‐x_CoO_3‐δ_ were synthesized as anode materials. The optimized composition with in situ reduction can form CoFe alloy nanoparticles, showing superior anodic CH_4_ reforming performance, with a CH_4_ conversion of 86.9% and CO selectivity of 90.1% at 800°C. Moreover, stable operation over 1250 h with a CO selectivity above 95% is achieved (Figure 15d–g). Through utilizing this efficient catalyst, the electrical energy consumption for CO production decreases from 3.46 kWh m^−3^ for conventional SOERs to 0.31 kWh m^−3^ for CH_4_‐assisted SOERs. The characterizations and DFT calculations revealed that CH_4_ undergoes cracking on the nanoparticles to H_2_ and coke formation, followed by reaction with spillover oxygen species to form CO [27]. Similar results were obtained through in situ exsolve Ni‐Fe alloy from a composite catalyst of La_1.2_Sr_0.8_Fe_0.6_Mn_0.4_O_4‐δ_ Ruddlesden‒Popper oxide and Ni‐Ce_0.85_Sm_0.15_O_2‐δ_ fluorite oxides [231].

### 6.3 Oxidative Coupling of Methane

Converting methane into value‐added olefins is economically attractive. In methane oxidative coupling processes, CH_4_ is generally believed to undergo initial oxidative activation to form C_2_H_6_, which subsequently experiences further oxidative dehydrogenation to produce C_2_H_4_. The yields of C_2_H_4_ and C_2_H_6_ are constrained by secondary reactions, such as the recombination of ·CH_3_ radicals with the catalyst surface and the further oxidation of C_2_H_6_, both on the catalyst and in the gas phase. Furthermore, reactive surface oxygen species, such as O^−^ and O_2_^2−^, play critical roles in activating CH_4_, thus raising stringent requirements on the catalyst surface, which must continuously supply active oxygen species to sustain the reaction [232]. This requirement explains why conventional thermal OCM catalysts typically employ metal‐oxide catalysts, such as Li/MgO and Sn/Li/MgO, in which the oxide substrate serves as a reservoir of reactive oxygen species [233].

**FIGURE 16:**
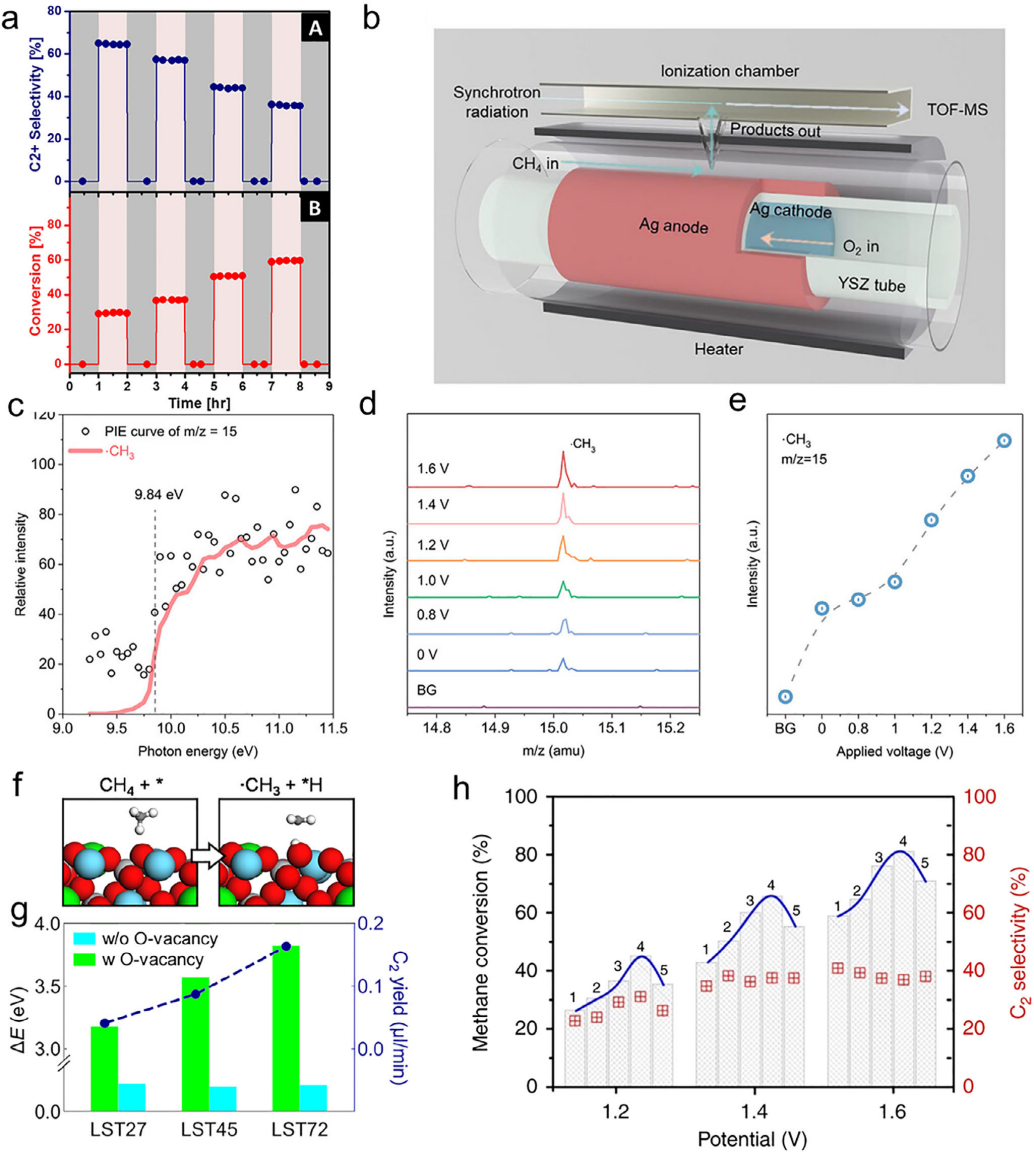
(a) C_2+_ selectivity and methane conversion as a function of reaction time and cell current. Catalyst: Mn‐Ce‐Na_2_WO_4_/SiO_2_ gel. Reproduced with permission [235]. Copyright 2017, Elsevier. (b) Diagram illustration of the in situ SVUV‐PIMS setup. (c) Experimental PIE curves and scaled photoionization cross section of mass‐to‐charge ratio (m/z) = 15. (d, e) Potential‐dependent ·CH_3_ intensity in PIMS of 10.6 eV. Reproduced with permission [19]. Copyright 2025, John Wiley and Sons. (f) Schematic diagram of the reaction mechanism for the methane activation step (CH_4_ + * → ·CH_3_ + *H) on the catalyst surface (side view). (g) Calculated reaction energy of the methane activation step (histogram) and experimental C_2_ yield (line chart) at different La/Sr ratios with and without O vacancies. La: large blue spheres; Sr: large green spheres; Ti: large gray spheres; O: large red spheres; C: small gray spheres; H: small white spheres; w/o: without; w: with. Reproduced with permission from [33], copyright 2024, Royal Society of Chemistry. h, CH_4_ conversion and C_2_ selectivity after cycling the output gas. 1: Sr_2_Fe_1.5_Mo_0.5_O_6−_ *_δ_*, 2: Sr_2_Fe_1.525_Mo_0.5_O_6‐δ_, 3: Sr_2_Fe_1.55_Mo_0.5_O_6‐_ *_δ_*, 4: Sr_2_Fe_1.575_Mo_0.5_O_6‐_ *_δ_*, and 5: Sr_2_Fe_1.6_Mo_0.5_O_6−_ *_δ_*. Reproduced with permission from [128], under the terms of Creative Commons CC BY license.

The O‐SOER can provide controllable oxygen species for methane oxidative reactions [234]. For instance, it was proven that methane conversion and C_2+_ selectivity can be effectively regulated by applying currents with an anode decorated with a Mn‐Ce‐Na_2_WO_4_/SiO_2_ catalyst (Figure 16a) [235]. The development of SVUV‐PIMS enables online capture of the active ·CH_3_ intermediates during methane oxidative coupling in an O‐SOER. In this study, commercial nanosized silver particles (60‐120 nm) served as both the anode and cathode (Figure 16b). The porous architecture of the Ag electrodes offers abundant diffusion channels for CH_4_ and ensures tight contact with the YSZ electrolyte. Upon applying an external bias, O^2−^ ions are transported from the electrolyte to the anode surface, where they are electrochemically activated into reactive oxygen species capable of dissociating hydrogen from CH_4_. This process generates transient ·CH_3_ radicals, whose signal intensity increases systematically with applied voltage (Figure 16c–e). The ·CH_3_ radicals subsequently desorb and undergo gas‐phase coupling to produce C_2_H_6_, which can further dehydrogenate to C_2_H_4_ [19].

To enhance economic attractiveness, it is critical to develop efficient and reliable catalysts [236, 237]. Tailoring surface chemistry, particularly the concentration of surface oxygen vacancies, has been demonstrated as an effective strategy to regulate methane activation pathways. For example, a series of La‐doped SrTiO_3_ perovskite oxides was synthesized as anode materials for O‐SOER, in which the La/Sr ratio at the A‐site was systematically varied to control the formation of oxygen vacancies and lattice oxygen species. Tuning this ratio directly altered the surface oxygen concentration and consequently affected the production of C_2_ hydrocarbons. Interestingly, the composition featuring the highest lattice oxygen content and the fewest oxygen vacancies exhibited the greatest C_2_ yield. DFT calculations verified these findings, revealing that methane activation is energetically more favorable on surfaces that are enriched with lattice oxygen and depleted in oxygen vacancies; such surfaces facilitate more efficient C‐H bond activation, leading to significantly higher conversion rates (Figure 16f,g) [33].

Beyond surface chemistry modulation, engineering the metal‐oxide interface has emerged as another powerful strategy to boost electrochemical activity and C_2_ hydrocarbon formation. For example, a porous scaffold electrode based on Sr_2_Fe_1.5+x_Mo_0.5_O_6‐δ_ with an in situ‐generated metal/oxide interface, delivering outstanding performance, achieving a C_2_ selectivity of 81.2% and a C_2_ concentration of 16.7% in the product, comprising 12.1% ethylene and 4.6% ethane while maintaining an impressive methane conversion of 41% in a single pass (Figure 16h). Moreover, the system exhibits remarkable durability, showing no discernible performance decay after 100 h of continuous high‐temperature operation and enduring 10 redox cycles without noticeable degradation. This approach demonstrates that finely tuned metal‐oxide interfaces not only promote methane activation and coupling pathways but also address common challenges such as carbon deposition and thermal instability [128]. Table 4 summarizes the performance of OCM in SOERs, which typically operate at temperatures of approximately 800°C. Such high operating temperatures mainly arise from the high stability of the methane molecule. As a result, future development of SOER‐based OCM should place greater emphasis on designing efficient catalysts capable of activating ·CH_3_ species at lower temperatures.

**TABLE 4:** Summary of reported results of oxidative coupling of methane using SOERs.

Total reaction	Anode	Electrolyte	Cathode	Anode reaction	Cathode reaction	Current density (A cm^−2^)	Operation temperature(°C)	Faradaic efficiency (%)	Olefin selectivity (%)	Stability for electrochemical activity (h)	Refs.
2CH_4_+CO_2_ = C_2_H_4_+H_2_+CO+H_2_O	Sr_2_Fe_1.5+x_Mo_0.5_O_6‐δ_	LSGM	Sr_2_Fe_1.5+x_Mo_0.5_O_6‐δ_	2CH_4_+O^2−^→C_2_H_4_+H_2_+H_2_O+2e^−^	CO_2_+2e^−^→CO+O^2−^	1@1.6 V	850	100	N/A	100	[128]
2CH_4_+CO_2_═C_2_H_4_+H_2_+CO+H_2_O	Ni‐CeO_2_	YSZ	Ni‐CeO_2_	2CH_4_+O^2−^→C_2_H_4_+H_2_+H_2_O+2e^−^	CO_2_+2e^−^→CO+O^2−^	1.2@1.6 V	800	90	N/A	100	[17]
2CH_4_+2O^2−^ = C_2_H_4_+2H_2_O+4e^−^	La_0.7_Sr_0.2_TiO_3‐δ_	GDC	LSCF/GDC	2CH_4_+2O^2−^→C_2_H_4_+2H_2_O+4e^−^	O_2_+4e^−^→2O^2−^	N/A	850	N/A	100	20	[33]
2CH_4_+2O^2−^ = C_2_H_4_+2H_2_O+4e^−^	BaMg_0.33_Nb_0.67‐x_Fe_x_O_3‐δ_	LSGM	SFM‐GDC	2CH_4_+2O^2−^→C_2_H_4_ +2H_2_O+4e^−^	O_2_+4e^−^→2O^2^	0.03@1.5 V	925	20	50.3	25	[34]
2CH_4_+2O^2−^ = C_2_H_4_+2H_2_O+4e^−^	Sr_2_Fe_1.575_Mo_0.5_O_6‐δ_	LSGM	Sr_2_Fe_1.5_Mo_0.5_O_6‐δ_	2CH_4_+2O^2−^→C_2_H_4_ +2H_2_O+4e^−^	O_2_+4e^−^→2O^2^	0.23@1.8 V	850	40	82	25	[35]
2CH_4_+2O^2−^ = C_2_H_4_+2H_2_O+4e	La_0.3_Sr_0.7_TiO_3+δ_	YSZ	Pt	2CH_4_+2O^2−^→C_2_H_4_ +2H_2_O+4e^−^	O_2_+4e^−^→2O^2^	0.028@9 V	850	N/A	5	N/A	[238]
2CH_4_+2O^2−^ = C_2_H_4_+2H_2_O+4e	Mn‐Ce‐Na_2_WO_4_/SiO_2_	ScSZ	LSM‐GDC	2CH_4_+2O^2−^→C_2_H_4_ +2H_2_O+4e^−^	O_2_+4e^−^→2O^2^	0.37@0.49 V	800	N/A	59.4	9	[235]
2CH_4_+2O^2−^ = C_2_H_4_+2H_2_O+4e	Sr_2_Fe_1.5_Mo_0.5_O_6‐δ_	ScSZ	Pr_6_O_11_	2CH_4_+2O^2−^→C_2_H_4_ +2H_2_O+4e^−^	O_2_+4e^−^→2O^2^	0.7@0.5 V	750	42.8	71.6	200	[239]

### 6.4 Nonoxidative Coupling of Methane

Nonoxidative coupling of methane (NOCM) is the direct transformation of methane into higher hydrocarbons without oxygen. Compared with OCM, NOCM is thermodynamically less favorable and prone to severe carbon deposition; however, it typically enables higher C_2_ selectivity under optimized conditions. Taking NOCM to ethene as an example, the reaction proceeds through three fundamental steps: (1) CH_4_ molecules are adsorbed onto the catalyst surface, (2) the C─H bond is activated and dissociated by catalytically active sites, producing surface‐bound methyl species (·CH_3_) and/or H_2_, and (3) adjacent surface methyl intermediates couple and subsequently desorb to form C_2_H_4_ and H_2_ in the gas phase [240]. Despite its potential, NOCM has received less attention in SOERs than OCM. The primary challenges arise from its inherently higher energy demand and the susceptibility to carbon deposition, as no oxygen species are available to remove deeply dehydrogenated carbonaceous intermediates.

The primary focus of NOCM research in SOERs lies in the design of catalysts with strong resistance to carbon deposition. An example of thermal catalytic NOCM was conducted over Pt/CeO_2_ catalysts and revealed that the key physicochemical steps consist of CH_4_ adsorption, the dissociation of the first H atom from surface‐bound CH_4_, and the subsequent adsorption/desorption of H_2_. Notably, the later C–C coupling steps contribute little to the overall CH_4_ conversion. Instead, the formation of C_2_ hydrocarbons is governed by Pt‐mediated CH_4_ adsorption‐desorption processes, along with the formation and desorption of C_2_ species and H_2_ from the catalyst surface. Although carbon deposition occurs during operation, the Pt/CeO_2_ catalyst maintained remarkable stability for 245 h. This durability is because coke deposits on the reactor walls rather than on the catalytic surface, thereby preserving active sites and avoiding activity loss [241].

**FIGURE 17:**
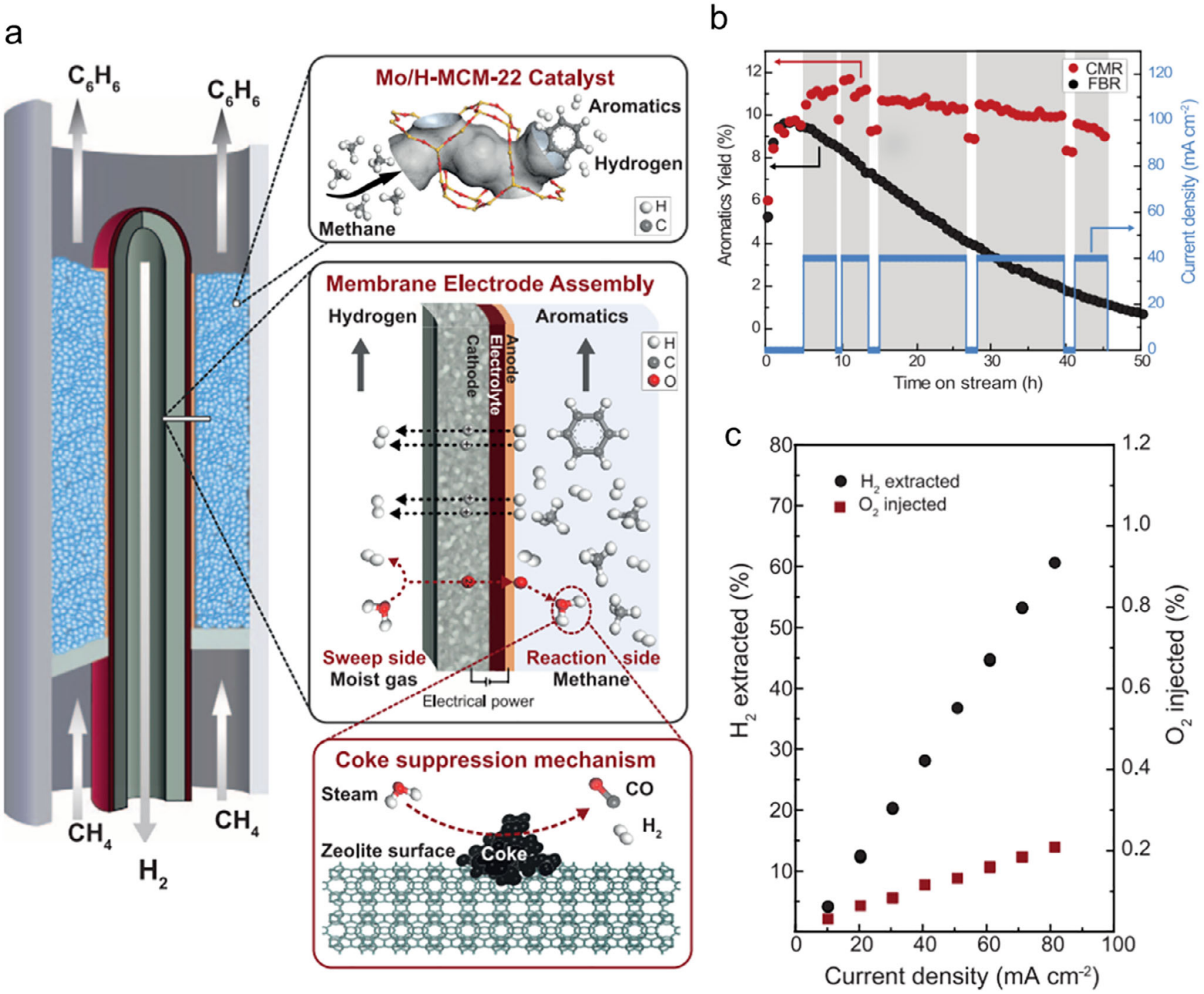
(a) CH_4_ is converted to benzene and hydrogen via a Mo/zeolite catalyst. H_2_ is transported as protons to the sweep side. Oxide ions are transported to the reaction medium to react with H_2_ and form steam as an intermediate before reacting with coke to form CO and H_2_. (b) Aromatic yield versus time. Gray‐shaded areas indicate when hydrogen is extracted. (c) Percentage of H_2_ extracted and O_2_ injected versus current density at 700°C. The anode is swept with a 10/90 mixture of H_2_/CH_4,_ and the cathode is swept with a 3/5/92 mixture of H_2_O/H_2_/Ar. Reproduced with permission [2]. Copyright 2016. The American Association for the Advancement of Science.

Due to the limited availability of oxygen species, NOCM in SOERs is particularly attractive for producing hydrocarbons with higher carbon numbers, such as benzene. Nonoxidative methane dehydroaromatization (MDA, 6CH_4_ ↔ C_6_H_6_ + 9H_2_) was demonstrated using shape‐selective Mo/zeolite catalysts integrated into BaZrO_3_‐based SOERs (Figure 17a). Acceptor‐doped BaZrO_3_ electrolytes function as proton conductors at intermediate temperatures and can partially conduct oxide ions at elevated temperatures. Compare to a fixed‐bed reactor (FBR) using 6Mo/MCM‐22 as the catalyst. The aromatic yield declined sharply as the reaction proceeded. In contrast, when an electrical current was applied to the SOER, the aromatics yield continued to rise and reached a maximum of ∼12% before catalyst activity began to decrease. The system exhibited an almost instantaneous catalytic response to current on‐off switching and to variations in current density, enabling precise, real‐time tuning of reaction performance (Figure 17b). The yield enhancements originate from the concurrent extraction of H_2_ and controlled oxide ion conduction along the reactor channel. Furthermore, the elevated operating temperature at 700°C enables the mixed conduction of oxygen ions and protons, thereby reducing carbon deposition (Figure 17c). This SOER realizes exceptionally high carbon utilization efficiencies up to 80%, significantly improving the techno‐economic viability of the MDA process [2].

### 6.5 Alkane Dehydrogenation to Olefins

In addition to methane, other alkanes, such as ethane and propane, have also been used for dehydrogenation to olefins (e.g., ethene, propylene) in SOERs. Similar to methane conversion, alkane dehydrogenation can be classified into oxidative and nonoxidative routes [86]. The former is employed in O‐SOERs, while the latter is mainly used in P‐SOERs. The dehydronated protons can be recombined at the cathode to form high‐purity hydrogen, enabling the coproduction of hydrogen and olefins in a P‐SOER. For example, a highly efficient La/Ni codoped strontium titanate (La_0.3_Sr_0.7_)_0.9_Ni_0.1_Ti_0.9_O_3‐δ_ (LSNT) perovskite catalyst has been developed and integrated into a P‐SOER with BaZr_0.8_Y_0.2_O_3_ as the electrolyte using commercial propane as the feedstock. Propane conversion and hydrogen production are significantly enhanced under applied current because of the electrochemical promotion effect and the shifted reaction equilibrium driven by rapid hydrogen removal. The introduction of water vapor into the feed further improves catalyst stability by suppressing coke deposition. Under a current density of 90 mA·cm^−2^ at 600°C, a propane conversion of up to 53% can be achieved. Furthermore, the LSNT catalyst demonstrates excellent tolerance to sulfur impurities that are commonly present in commercial propane. The superior performance is attributed to the formation of highly active and selective Ni species at the perovskite‐nano nickel particle interface, which are generated in situ through chemical reduction under operating conditions [242].

**FIGURE 18:**
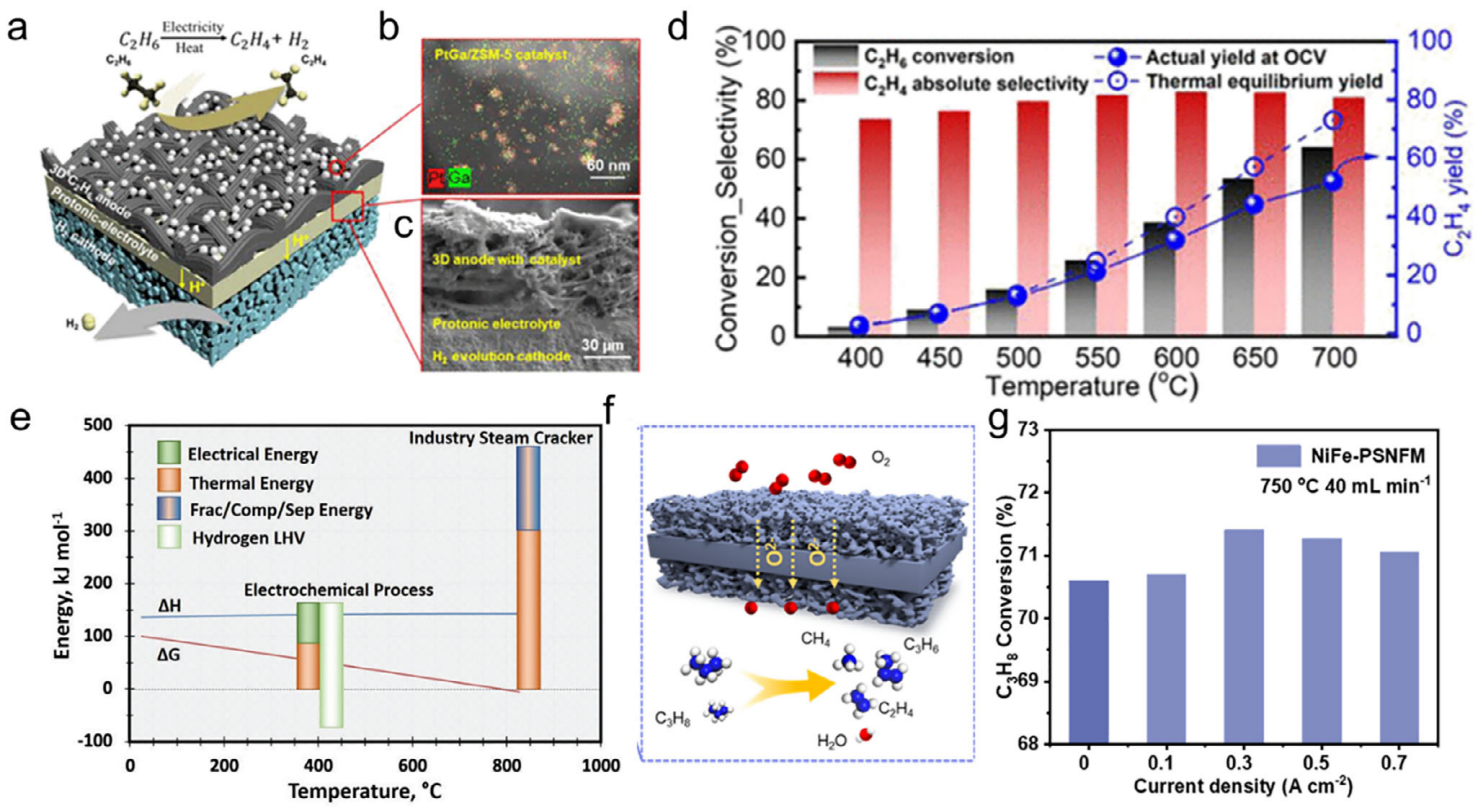
(a) Schematic of the ethane dehydrogenation process in a proton‐conducting electrochemical cell with a 3D fuel electrode. Ethane was fed into the anode and dehydrogenated to ethylene and protons, which were transported through the electrolyte membrane to the cathode and combined with electrons to form hydrogen. (b) Scanning TEM image of the as‐prepared PtGa/ZSM‐5 catalyst loaded in the 3D anode before testing. (c) Cross‐sectional SEM image of the catalyst‐integrated electrochemical cell. (d) Performance at different temperatures under OCV conditions. Reproduced with permission from [243], copyright 2021, American Chemical Society. (e) Comparison of the process energies for ethylene production from ethane between electrochemical processes and industrial steam crackers. Reproduced with permission from [25], copyright 2018. Royal Society of Chemistry. (f) Schematic illustration of the cogeneration of light olefins and electricity via oxide‐ion‐conducting SOERs. (g) C_3_H_8_ conversion as a function of current density. Reproduced with permission [26]. Copyright 2023, American Chemical Society.

It is worth emphasizing that the integration of efficient catalysts into SOERs can lead to a low energy budget for chemical synthesis. An example is the production of hydrogen and ethylene from ethane, which uses a planar P‐SOER equipped with a bifunctional three‐dimensional catalytic electrode (Figure 18 a‐c). A single‐pass ethane conversion of 40% and an ethylene yield of 26.7% are achieved at 550°C (Figure 18d). Compared with conventional industrial ethane steam cracking, this electrochemical approach reduces the process energy input by 45.1% and enhances the overall energy efficiency by 50.6%. Furthermore, steam electrolysis operated in solid oxide electrolysis cell mode can regenerate the catalytic performance and mitigate catalytic degradation by 74%, highlighting the high techno‐economic viability of this technology [243]. Similarly, a P‐SOER comprising PrBa_0.5_Sr_0.5_Co_1.5_Fe_0.5_O_6‐δ_ was used as the anode, Ni‐BZCYYb was used as the cathode, and BZCYYb was used as the electrolyte for ethane dehydrogenation. When operated at 400°C and a constant current density of 1 A cm^−2^, the P‐SOER achieved nearly 100% selectivity toward ethylene at an electrochemical overpotential of only 140 mV. Compared with a conventional industrial ethane steam cracker, the electrochemical dehydrogenation route can reduce process energy consumption by approximately 65% and lower the carbon footprint by up to 72%. Moreover, the electrical energy required for the electrochemical dehydrogenation process can be offset by the heating value of the coproduced hydrogen, enabling the overall system to operate with a low energy budget and at substantially lower energy consumption and operating temperatures than conventional industrial steam cracking (Figure 18e) [25]. To remove carbon deposition, the catalysts should possess the capability of abundant oxygen species. The metal oxide material Ce_0.9_(Ni_x_Cu_1‐x_)_0.1_O_2‐δ_ was developed for ethane dehydrogenation in SOER, and the prereduction forms a Ni‐Cu alloy on the CeO_2_ surface. The current density reaches 0.51 A cm^−2^ at 700°C and 1.0 V, and the ethane conversion rate is 38.9% with an ethylene yield of 24.9%, demonstrating outstanding stability even after being operated continuously for a period of one hundred hours [244].

Alkane oxidative dehydrogenation to olefins using O‐SOER represents another important pathway to produce value‐added products. Uniquely, alkane oxidative dehydrogenation is exothermic, and it is possible to achieve thermal neutrality when combined with CO_2_ and H_2_O electrolysis. However, achieving both a high alkane conversion rate and high ethylene selectivity remains challenging. One effective strategy is to combine compositional doping with electrochemical activation to overcome the conventional activity‐selectivity trade‐off and achieve a high ethylene yield. In a study using Sr_2_Ti_0.8_(Co_1.2‐x_Fe_x_)O_6‐δ_ as model electrodes with varying dopant ratios, it was shown that increasing the Fe content effectively reduces the oxygen activity by weakening the metal‐oxygen covalency, downshifting the O 2p band relative to the Fermi level, and increasing the oxygen vacancy formation energy. These modifications lead to a lower ethane conversion rate but higher ethylene selectivity for Sr_2_Ti_0.8_Fe_1.2_O_6‐δ_ (STF) compared to electrodes with higher Co content. By increasing the applied potential, the ethane conversion rate can be enhanced without significantly compromising ethylene selectivity. Ultimately, the SOEC with an STF anode achieves an ethylene yield of up to 71% at 800°C and 1.2 V [245]. Another example is an active electrode with an in situ grown metal‐oxide interface that significantly promotes the activation of the ethane C‐H bond, leading to efficient ethylene production. Under the coelectrolysis mode of ethane with CO_2_, the Co@CeO_2_ electrode demonstrates exceptional performance, achieving an ethane conversion rate of 33.1% and an ethylene selectivity of 88.9% at an applied voltage of 1.0 V. Moreover, the metal‐oxide interface constructed via an in situ exsolved method effectively prevents the agglomeration of nanoparticles at high temperatures; notably, the electrode's performance does not exhibit significant degradation even after 100 h of electrochemical reaction, which is attributed to the regulation of oxygen species in O‐SOER for carbon removal [246].

The coproduction of electricity and olefins can be achieved in both the P‐SOER and O‐SOER when the anode is fed light alkanes while the cathode is exposed to air to build a high chemical potential. For example, an efficient catalyst is NiFe alloy nanoparticles (NPs) exsolved from a Pr_0.8_Sr_1.2_Ni_0.2_Fe_1.3_Mo_0.5_O_6‑δ_ (PSNFM) matrix during O‐SOER operation. It was found that Ni is first exsolved, which triggers the following Fe‐exsolution, forming the NiFe NP alloy. The surface reconstruction process creates abundant oxygen vacancies at the NiFe/PSNFM interface, which promotes oxygen mobility for the oxidative dehydrogenation of propane and enhances coking resistance. At 750°C, the SOER reactor with the PSNFM catalyst reaches a propane conversion of 71.40% and light olefin yield of 70.91% under a current density of 0.3 A cm^−2^ without coking (Figure 18f,g) [26]. In terms of P‐SOERs, the removal of protons from propane enables the cogeneration of olefins and electricity. Previous studies have demonstrated this concept by developing a (Pr_0.3_Sr_0.7_)_0.9_Ni_0.1_Ti_0.9_O_3_ (PSNT) catalyst layer over a P‐SOER anode. Under reducing conditions, PSNTs undergo surface reconstruction and exsolve Ni nanoparticles in situ, creating additional active sites and inhibiting carbon deposition to enhance durability. However, the addition of 10% H_2_O is still required to suppress carbon deposition during operation. The P‐SOER exhibited increasing selectivity toward propylene and ethylene with increasing current density, reaching 36% and 32%, respectively, at 300 mA·cm^−2^ and 700°C [72]. Table 5 summarizes alkane dehydrogenation in the O‐SOER and P‐SOER. It is found that ethane dehydrogenation to ethene exhibits higher selectivity and can operate at lower temperatures. However, for alkanes with more carbon atoms, such as propane, side reactions occur and lead to lower propene selectivity. Future studies should focus on carefully tailoring catalyst performance and lowering the operating temperature to enhance propene selectivity, as it is more economically attractive than ethene [86].

**TABLE 5:** Summary of alkane dehydrogenation to olefins using SOERs.

Total reaction	Anode	Electrolyte	Cathode	Anode reaction	Cathode reaction	Current density (A cm^−2^)	Operation temperature (°C)	Faradaic efficiency (%)	Olefin selectivity (%)	Stability for electrochemical activity (h)	Refs.
C_3_H_8_+CO_2_ = C_3_H_6_+ CO +H_2_O	(La_0.3_Sr_0.7_)_0.9_(Ti_0.85_Mn_0.15_)_0.9_Ni_0.1_O_3+_ *_δ_*	YSZ	(La_0.3_Sr_0.7_)_0.9_(Ti_0.85_Mn_0.15_)_0.9_Ni_0.1_O_3+_ *_δ_*	C_3_H_8_+O^2−^→C_3_H_6_+H_2_O+2e^−^	CO_2_+2e^−^→CO+O^2−^	0.55@1.6 V	700	88	37.5	0.5	[247]
C_2_H_6_+CO_2_ = C_2_H_4_+CO+H_2_O	Sr_2_Ti_0.8_Co_0.6_Fe_0.6_O_6‑δ_	LSGM	Sr_2_Ti_0.8_Co_0.6_Fe_0.6_O_6‑δ_	C_2_H_6_+O^2−^→C_2_H_4_+H_2_O+2e^−^	CO_2_+2e^−^→CO+O^2−^	0.3@1.5 V	800	90	N/A	100	[248]
C_2_H_6_+CO_2_ = C_2_H_4_+CO+H_2_O	CeO_2_	YSZ	CeO_2_	C_2_H_6_+O^2−^→C_2_H_4_+H_2_O+2e^−^	CO_2_+2e^−^→CO+O^2−^	0.6@1.6 V	600	82	N/A	300	[18]
C_2_H_6_+CO_2_ = C_2_H_4_+CO+H_2_O	LSCF‐SDC+Al_2_O_3_	LSGM	LSCF‐SDC	C_2_H_6_+O^2−^→C_2_H_4_+H_2_O+2e^−^	CO_2_+2e^−^→CO+O^2−^	NA	600	100	N/A	200	[249]
C_2_H_6_+CO_2_ = C_2_H_4_+CO+H_2_O	Ni*_x_* Cu_1–_ *_x_*‐doped Nb_1.33_(Ti_0.8_Mn_0.2_)_0.67_O_4‐δ_	BZCYYb1711	NiO‐BCZYYb	C_2_H_6_→C_2_H_4_+2H^+^+2e^−^	CO_2_+2H^+^+2e^−^→CO+H_2_O	0.8@0.8 V	700	100	N/A	10	[250]
C_3_H_8_+0.5O_2_ = C_3_H_6_+H_2_O	(La_0.8_Sr_0.2_)_0.95_MnO_3_‐ SrCe_0.95_Yb_0.05_O_3_	YSZ	(La_0.8_Sr_0.2_)_0.95_MnO_3_‐ SrCe_0.95_Yb_0.05_O_3_	C_3_H_8_+O^2−^→C_3_H_6_+H_2_O+2e^−^	O_2_+4e^−^→2O^2^	0.01@1 V	600	N/A	58	3	[251]
C_3_H_8_ = C_3_H_6_+H_2_	(La_0.3_Sr_0.7_)_0.9_Ni_0.1_Ti_0.9_O_3‐δ_	BZY20	Ni‐BZCYYb1711	C_3_H_8_→C_3_H_6_+2H^+^+2e^−^	2H^+^+2e^−^→H_2_	0.09@1 V	600	N/A	N/A	45	[242]
C_2_H_6_ = C2H_4_+H_2_	PrBa_0.5_Sr_0.5_Co_1.5_Fe_0.5_O_6_	BZCYYb1711	Ni‐BZCYYb1711	C_2_H_6_→C_2_H_4_+2H^+^+2e^−^	2H^+^+2e^−^→H_2_	1@0.41 V	400	N/A	100	90	[25]
C_2_H_6_ = C_2_H_4_+H_2_	(3D) ultra‐porous (PrBa)_0.95_(Fe_0.9_Mo_0.1_)_2_O_5+δ_	BZCYYb1711	Ni‐BZCYYb1711	C_2_H_6_→C_2_H_4_+2H^+^+2e^−^	2H^+^+2e^−^→H_2_	0.17@1.4 V	600	N/A	82	150	[243]
C_2_H_6_ = C_2_H_4_+H_2_	Sr_3_Fe_1.7_Ni_0.05_Cu_0.05_Hf_0.2_O_7‐δ_	BZCYYb1711	Ni‐BZCYYb1711	C_2_H_6_→C_2_H_4_+2H^+^+2e^−^	2H^+^+2e^−^→H_2_	1.4@1.8 V	700	N/A	[90]	(1)	[252]
C_3_H_8_+0.5O_2_ = C_3_H_6_+H_2_O	(Pr_0.3_Sr_0.7_)_0.9_Ni_0.1_Ti_0.9_O_3‐δ_ ‐Ni‐BZCY	BZCY	LSCF‐BZCY	C_3_H_8_→C_3_H_6_+2H^+^+2e^−^	2H^+^+2e^−^+0.5O_2_→H_2_O	0.7@0.7 V	700	N/A	[50]	[50]	[72]
C_2_H_6_+0.5O_2_ = C_2_H_4_+H_2_O	SrMo_0.8_Co_0.1_Fe_0.1_O_3‐δ_	BZCY172	LSCF‐ BZCY172	C_2_H_6_→C_2_H_4_+2H^+^+2e^−^	2H^+^+2e^−^+0.5O_2_→H_2_O	0.8@1 V	750	N/A	[91]	[50]	[253]
C_2_H_6_+0.5O_2_ = C_2_H_4_+H_2_O	La_0.6_Sr_0.4_Fe_0.8_Nb_0.1_Cu_0.1_O_3−δ_	BZCYYb	La_0.6_Sr_0.4_Fe_0.8_Nb_0.1_Cu_0.1_O_3−δ_	C_2_H_6_→C_2_H_4_+2H^+^+2e^−^	2H^+^+2e^−^+0.5O_2_→H_2_O	0.3@0.8 V	750	N/A	[95]	[40]	[254]

*Note*: LSGM: La_0.8_Sr_0.2_Ga_0.8_Mg_0.2_O_3‐δ_; YSZ: yttria‐stabilized zirconia; LSCF: La_0.6_Sr_0.4_Co_0.2_Fe_0.8_O_3‐δ_; SDC: Sm_0.2_Ce_0.8_O_2‐δ_; BZCYYb1711: BaZr_0.1_Ce_0.7_Y_0.1_Yb_0.1_O_3‐δ_; BZY20: BaZr_0.8_Y_0.2_O_3‐δ_; LSM: (La_0.8_Sr_0.2_)_0.95_MnO_3‐δ_; BSCF: Ba_0.5_Sr_0.5_Co_0.8_Fe_0.2_O_3‐δ_; GDC: Gd_0.2_Ce_0.8_O_1.9_; BZCY: BaZr_0.3_Ce_0.5_Y_0.2_O_3‐δ_; BZCY172: BaCe_0.7_Zr_0.1_Y_0.2_O_3‐δ_; ScSZ: Scandium‐stabilized zirconia.

## 7 Application of AI for Materials Discovery and Performance Prediction in SOERs

In recent years, AI has emerged as a powerful tool for discovering efficient catalysts and predicting the electrochemical performance of SOERs, owing to its relatively low computational cost and high predictive accuracy. A variety of AI approaches have been employed for materials design and screening, including machine learning (ML) and deep learning (DL), as well as optimization techniques such as Bayesian optimization (BO), Monte Carlo simulation (MCS), genetic algorithms (GA), and particle swarm optimization (PSO) [21, 255]. For electrolyte discovery, Hyodo et al. developed a gradient‐augmented regression machine learning model incorporating 80 descriptors for perovskite oxides and constructed a hydration map as a powerful tool to screen 8,613 oxide candidates. The predicted material, BaSn_0.8_Sc_0.2_O_3‐δ_, exhibited a proton conductivity of 1.4 × 10^−3^ S cm^−1^ at 380°C [256]. In another study, Luo et al. employed high‐throughput computations to evaluate the oxygen vacancy formation energy, hydration energy, and adsorption energies of H_2_O and CO_2_ across 932 oxides. Their results identified BaSn_x_Ce_0.8‐x_Yb_0.2_O_3‐δ_ as a promising electrolyte, which was experimentally validated to enable stable SOER operation for 1,000 h under 50% H_2_O steam conditions [257]. Beyond electrolytes, AI has also been applied to the design of electrode materials. For example, researchers have used ionic Lewis acid strength as a descriptor in artificial neural network models to predict oxygen reduction reaction activity in oxides. The screened material, Sr_0.9_Cs_0.1_Co_0.9_Nb_0.1_O_3‐δ_, demonstrated low polarization resistance and stable operation for over 800 h [20].

In addition to materials development, AI technologies have also been applied to predict the electrochemical performance and stability of SOER [258, 259, 260]. The evolution of nickel and nickel oxide during SOER operation is a key factor governing device stability. Sciazko et al. proposed an unsupervised image‐to‐image translation (UNIT) network framework incorporating physical constraints to predict the reduction behavior of NiO in Ni‐based fuel electrodes. The results demonstrate that the UNIT model can successfully predict the NiO reduction process and can potentially be extended to other microstructural evolutions, such as Ni migration and particle coarsening under high current density and humid operating conditions [261]. In addition, the electrochemical performance of SOER systems has been predicted using machine learning models based on the extreme gradient boosting (XGBoost) algorithm, and the analysis revealed that effective area, operating voltage, and temperature are the most influential factors, contributing 21.6%, 16.6%, and 13.0%, respectively. The model further suggests that high temperature, elevated pressure, and low effective area are favorable conditions for achieving higher hydrogen production rates [258].

Although the application of AI in advancing SOER technologies is highly attractive, its effectiveness strongly depends on the quality and consistency of the underlying datasets. Notably, different research groups often report scattered or inconsistent results, even for similar materials, cell configurations, and operating conditions. Such variability can have a detrimental impact on the reliability and generalizability of AI‐based predictions. Therefore, greater emphasis should be placed on generating high‐quality, standardized experimental data. As a result, the integration of automated and robotic experimental platforms is expected to play a crucial role in ensuring data reliability and reproducibility.

## 8 Outlooks

**FIGURE 19:**
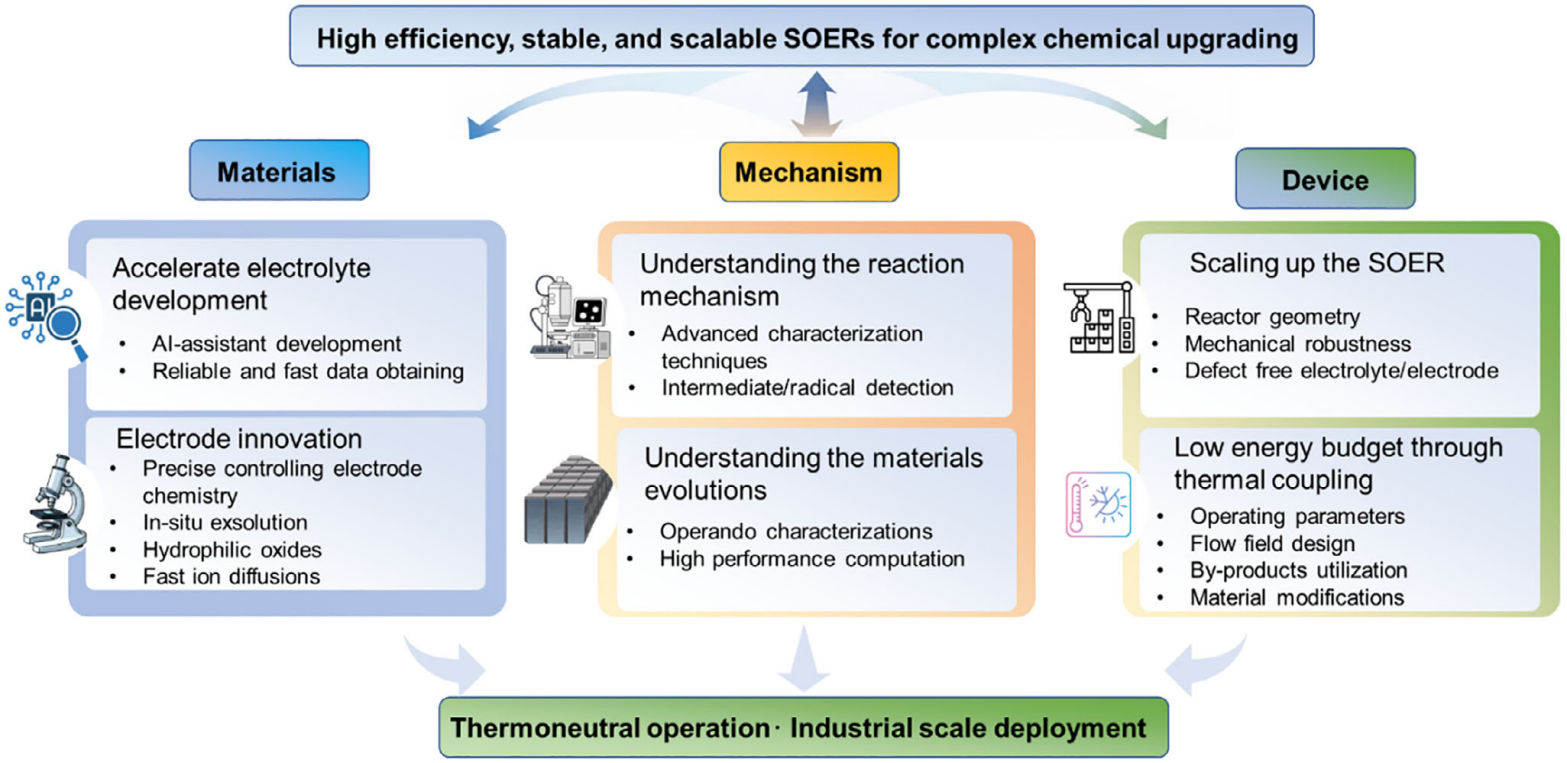
Outlooks for future SOER development.

To further promote the applications of SOER for chemical synthesis and better perform experiments in laboratory studies, several research areas should be carefully considered (Figure 19), including materials innovation, mechanism understanding, and process optimization. From an engineering perspective, laboratory studies face substantial challenges in accurately determining product distributions and production rates under varying temperatures and current densities, particularly when using small button cells. Effective experimentation requires careful control of SOER sealing quality, as well as precise and timely detection of outlet gas compositions and subtle changes in gas flow rates between the inlet and outlet streams. Moreover, it should be noted that solid carbon deposits and liquid byproducts are often generated in SOERs during hydrocarbon electrochemical reactions, which further complicates product analysis and quantification. Enlarging the SOER size is an effective strategy to address these challenges [262, 263]; however, it introduces significant engineering difficulties in controlling reactor geometry, ensuring mechanical robustness, and achieving uniform, defect‐free electrode and electrolyte layers. Although substantial progress has been made over the past decades in the fabrication of large‐area SOERs, these developments have predominantly focused on zirconia‐based O‐SOERs employing nickel‐based composite electrodes. Their applications have largely been limited to relatively simple reactions, such as hydrogen‐fueled fuel cells and H_2_O and CO_2_ electrolysis [264], while studies involving large‐scale SOERs for more complex chemical reactions remain limited.

Second, coupling two reactions at the two electrodes of an SOER offers a critical advantage by enabling the integration of an endothermic reaction (e.g., CO_2_ electrolysis) with an exothermic reaction (e.g., alkane oxidation) within a single electrochemical device. This unique configuration provides the potential to achieve thermoneutral operation through precise control of reaction kinetics, thereby significantly reducing the overall energy consumption of the SOER system. However, experimental verification of thermoneutral operation is rarely achieved in small‐scale SOERs, necessitating system‐level considerations based on large devices. These include optimization of operating parameters, flow‐channel and flow‐field design, integration of independent cooling systems, materials modification, and innovations in balance‐of‐plant components. At the device level, careful regulation of the extent of the anodic and cathodic reactions through applied current control is essential. Furthermore, the effective utilization of byproducts, such as generating the required heat by combusting hydrogen produced during alkane dehydrogenation, provides an additional pathway toward low energy budgets; however, this strategy requires SOERs with high electrochemical performance operating at low energy consumption.

To further enhance electrochemical performance and stability, reducing the operating temperature is critical, as many side reactions, such as carbon deposition, electrode poisoning, and electrode evolution, are thermodynamically and kinetically favored at elevated temperatures. Developing efficient electrolytes represents the most feasible strategy for lowering operating temperatures and should simultaneously address high ionic conductivity, large ionic transference numbers, and long‐term stability under harsh operating conditions. Conventional trial‐and‐error approaches are increasingly insufficient to meet the urgent demand for electrolyte innovation. Thus, the utilization of AI‐assisted methodologies has emerged as a promising route to accelerate electrolyte discovery and optimization, particularly given that electrolytes generally operate under simpler and more well‐defined conditions than electrode materials. Indeed, recent studies have demonstrated the substantial potential of AI‐based approaches in expediting electrolyte development [16, 265, 266]. Furthermore, advances in fabrication processes that enable precise control over large‐area electrolyte films, together with a thorough understanding of electrolyte operational limitations, are also critical for the practical deployment of SOER technologies.

In terms of electrode materials, they must exhibit high electrocatalytic activity while maintaining sufficient electrical conductivity under specific reaction environments. The development of heterogeneous catalysts has been proven effective. However, current fabrication strategies face limitations in precisely controlling heterogeneous interfaces. In situ exsolution can compromise the structural integrity of the host lattice, whereas postinfiltration methods suffer from nanoparticle coarsening during operation. Future developments should put more effort into precisely controlling the surface chemistry, revealing the effects of the functions of each competent oxide and their interface, and capturing the surface evolution process online.

With respect to elucidating reaction mechanisms in SOERs, a detailed understanding of the coupling between electrochemical and chemical reactions is of particular importance, especially for catalyzing complex processes such as alkane oxidation. Although previous studies have successfully achieved online detection of free radicals during SOER‐based methane coupling, most of these investigations were conducted under low‐pressure conditions, which differ significantly from practical operating environments. Achieving this goal requires the development of advanced characterization techniques capable of capturing key reaction intermediates, together with high‐performance computational methods to propose and validate reaction pathways.

## 9 Conclusion

In this review, we provide a comprehensive summary of SOERs for chemical upgrading. We systematically examine both O‐SOERs and P‐SOERs, with particular emphasis on the key factors influencing their efficiency and stability. The low efficiency of SOERs is primarily attributed to electrolytes with low ionic transference numbers and insufficient electrode performance. A thorough understanding of the operational limitations of each electrolyte type, together with full utilization of their intrinsic properties, is essential for achieving high efficiency. In terms of stability, carbon deposition, sulfur poisoning, electrode surface reconstruction, and electrolyte degradation are identified as the major causes of performance deterioration. From a thermodynamic perspective, regulating operating conditions, including temperature, reactant composition, and operating pressure, has proven effective in prolonging device lifetime. From a materials perspective, the development of electrodes featuring mobile oxygen species, enhanced hydrophilicity, and strategically exsolved nanoparticles has been demonstrated to significantly improve performance and operational stability. Owing to their distinct operating temperature windows, O‐SOERs and P‐SOERs exhibit unique advantages in specific reactions. Accordingly, we review their applications in CO_2_ reduction, CO_2_ reduction coupling with F‐T synthesis, the production of value‐added hydrocarbons via anodic alkane oxidative coupling, and the cogeneration of electricity and olefins. Finally, recent advances in electrode design aimed at addressing these challenges and further enhancing SOER performance are summarized.

## Funding

This work was supported by the Australian Research Council Discovery Projects (grant no. DP200103315, DP200103332 and DP230100685) and Australian Research Council Industrial Transformation Research Hubs (grant no. IH220100012).

## Conflicts of Interest

The authors declare no conflicts of interest.

## Data Availability

The data that support the findings of this study are available from the corresponding author upon reasonable request.
